# Ultraviolet stress delays chromosome replication in light/dark synchronized cells of the marine cyanobacterium *Prochlorococcus marinus *PCC9511

**DOI:** 10.1186/1471-2180-10-204

**Published:** 2010-07-29

**Authors:** Christian Kolowrat, Frédéric Partensky, Daniella Mella-Flores, Gildas Le Corguillé, Christophe Boutte, Nicolas Blot, Morgane Ratin, Martial Ferréol, Xavier Lecomte, Priscillia Gourvil, Jean-François Lennon, David M Kehoe, Laurence Garczarek

**Affiliations:** 1 UPMC-Université Paris 06, Station Biologique, Place Georges Teissier, 29680 Roscoff, France; 2 CNRS, UMR 7144, Groupe Plancton Océanique, 29680 Roscoff, France; 3 CNRS, FR 2424, Service Informatique et Génomique, 29680 Roscoff, France; 4 Clermont Université, Université Blaise Pascal, UMR CNRS 6023, Laboratoire Microorganismes: Génome et Environnement, BP 10448, 63000 Clermont-Ferrand, France; 5 CEMAGREF, UR Biologie des Ecosystèmes Aquatiques, Laboratoire d'Hydroécologie Quantitative, 3 bis quai Chauveau, CP 220, 69336 Lyon Cedex 09, France; 6 Department of Biology, 1001 East Third Street, Indiana University, Bloomington, IN 47405, USA

## Abstract

**Background:**

The marine cyanobacterium *Prochlorococcus *is very abundant in warm, nutrient-poor oceanic areas. The upper mixed layer of oceans is populated by high light-adapted *Prochlorococcus *ecotypes, which despite their tiny genome (~1.7 Mb) seem to have developed efficient strategies to cope with stressful levels of photosynthetically active and ultraviolet (UV) radiation. At a molecular level, little is known yet about how such minimalist microorganisms manage to sustain high growth rates and avoid potentially detrimental, UV-induced mutations to their DNA. To address this question, we studied the cell cycle dynamics of *P. marinus *PCC9511 cells grown under high fluxes of visible light in the presence or absence of UV radiation. Near natural light-dark cycles of both light sources were obtained using a custom-designed illumination system (cyclostat). Expression patterns of key DNA synthesis and repair, cell division, and clock genes were analyzed in order to decipher molecular mechanisms of adaptation to UV radiation.

**Results:**

The cell cycle of *P. marinus *PCC9511 was strongly synchronized by the day-night cycle. The most conspicuous response of cells to UV radiation was a delay in chromosome replication, with a peak of DNA synthesis shifted about 2 h into the dark period. This delay was seemingly linked to a strong downregulation of genes governing DNA replication (*dnaA*) and cell division (*ftsZ*, *sepF*), whereas most genes involved in DNA repair (such as *recA*, *phrA*, *uvrA*, *ruvC*, *umuC*) were already activated under high visible light and their expression levels were only slightly affected by additional UV exposure.

**Conclusions:**

*Prochlorococcus *cells modified the timing of the S phase in response to UV exposure, therefore reducing the risk that mutations would occur during this particularly sensitive stage of the cell cycle. We identified several possible explanations for the observed timeshift. Among these, the sharp decrease in transcript levels of the *dnaA *gene, encoding the DNA replication initiator protein, is sufficient by itself to explain this response, since DNA synthesis starts only when the cellular concentration of DnaA reaches a critical threshold. However, the observed response likely results from a more complex combination of UV-altered biological processes.

## Background

Since its discovery two decades ago [1], the marine cyanobacterial genus *Prochlorococcus *has rapidly become established as a model organism in microbial ecology [2-4]. As for other cyanobacteria with an obligate photoautotrophic lifestyle, *Prochlorococcus *has an absolute dependency on solar energy for cell maintenance and multiplication [5]. In the field, the rhythmic nature of light availability imposes a synchronization of its whole metabolism. Indeed, light/dark (L/D) entrained *Prochlorococcus *cells were shown to display a strong diurnal periodicity of many cellular functions, including cell cycle [6-8], pigment synthesis [9], carbon fixation [10], and amino acid uptake [11]. Synchronization primarily acts on gene expression, as evidenced first by studies focusing on individual cell cycle (e.g. *dnaA*, *ftsZ*) and photosynthesis related genes (e.g. *pcbA*, *psbA*) [12,13], then more recently at the whole transcriptome level [14]. Under optimal growth conditions, generation times of *Prochlorococcus *populations are generally around 24 h, though faster growth rates have sometimes been reported [8]. The DNA replication period is usually restricted to the late afternoon and dusk period and cytokinesis occurs during the night [6,7,13].

Studying the interplay between energy source fluctuations (i.e. changes in light quantities and/or spectral composition) and cell cycle dynamics of *Prochlorococcus *is of special interest as it lays the foundation for designing reliable population growth models for this key organism, considered to be the most abundant free-living photosynthetic organism on Earth [15]. As early as 1995, Vaulot and coworkers [7] noticed that in field populations of *Prochlorococcus*, the timing of DNA replication varied with depth, with the initiation of DNA synthesis occurring about 3 h earlier below the thermocline than in the upper mixed layer. At that time, these authors interpreted this delay as a possible protective mechanism to prevent exposure of replicating DNA to the high midday irradiances and especially UV. Since then, a number of studies have shown that *Prochlorococcus *populations are in fact composed of several genetically distinct ecotypes adapted to different light niches in the water column [16-18]. The upper mixed layer is dominated by the so-called high light adapted (HL) ecotypes (HLI and HLII, also called eMED4 and eMIT9312, respectively), whereas low light adapted (LL) ecotypes (such as LLII and LLIV, also called eSS120 and eMIT9313, respectively) are restricted to the bottom of the euphotic zone [19-22]. These studies also showed that a third ecotype (eNATL), initially classified as a LL clade (LLI), preferentially lived at intermediate depth, reaching maximal concentrations in the vicinity of the thermocline. Comparative genomics revealed that these various ecotypes display a number of genomic differences, including distinct sets of genes involved in DNA repair pathways [3,23,24]. For instance, genes encoding DNA photolyases, which are involved in the repair of thymidine dimers, are found in HL and eNATL ecotypes, but not in "true" LL strains (i.e., LLII-IV clades). Besides this light niche specialization, a dramatic genome reduction has affected all *Prochlorococcus *lineages except the LLIV clade, situated at the base of the *Prochlorococcus *radiation. This streamlining process seemingly reduced their signal transduction and gene expression regulatory capacity, raising the question how *Prochlorococcus *cells sense environmental signals and translate them into cellular responses [25]. Thus, HL ecotypes possess only five sensor histidine kinases and seven response regulators, the two protein types that make up two-component regulatory systems in cyanobacteria [4,24,26,27]. As this set is considerably smaller than that found in most other prokaryotes, additional regulatory mechanisms are likely to exist. Recent experimental evidence indeed suggested the involvement of sophisticated post-translational regulatory mechanisms and a key role of non-coding RNAs (ncRNAs) in acclimation processes of *Prochlorococcus marinus *MED4 cells to a variety of environmental stresses [28].

The discovery of ecotypes with different light response characteristics, each with a specific depth distribution in the field calls into question the abovementioned interpretation of the delay in DNA synthesis initiation noticed in field populations by Vaulot and coworkers [7]. Comparative cell cycle dynamics of the *P. marinus *HLI strain MED4 and the LLII strain SS120 under similar light/dark conditions indeed showed that SS120 initiated DNA replication 1-2 h earlier than MED4 [6]. So, ecotypic differences may also explain this delay. In the present paper, we reexamine this issue by directly characterizing the effects of UV radiation on the cell cycle dynamics and gene expression patterns of L/D synchronized cultures of the HLI strain PCC9511.

## Results

### Comparative cell cycle dynamics of acclimated *P. marinus* PCC9511 cells grown in batch cultures with and without UV radiation

**Figure 1:**
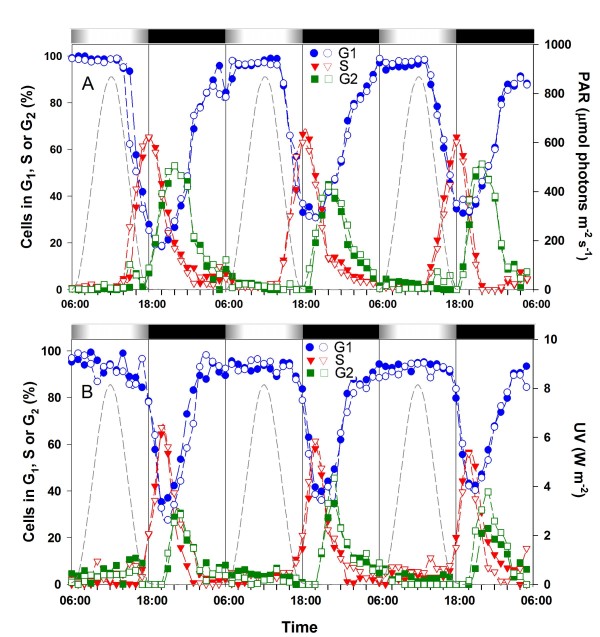
**Effect of UV exposure on the timing of the cell cycle phases of *Prochlorococcus marinus *PCC9511 cells grown over a 12 h/12 h light/dark cycle in batch culture**. **A**, distribution of cells in G_1 _(blue), S (red) and G_2 _(green) phases for batch cultures of PCC9511 grown under HL. **B**, same for HL+UV conditions. The experiment was done in duplicates shown by filled and empty symbols. Note that only the UV radiation curve is shown in graph B since the visible light curve is the same as in graph A. White and black bars indicate light and dark periods. The dashed line indicates the irradiance level (right axis). HL, high light; PAR, photosynthetically available radiation; UV, ultraviolet radiation.

A first series of preliminary experiments using batch cultures of *P. marinus *PCC9511 was performed in order to examine the effects of UV exposure on cell cycle and growth. Cells were acclimated for several weeks to a modulated 12 h/12 h L/D cycle of photosynthetically available radiation (PAR) reaching about 900 μmol photons m^-2 ^s^-1 ^at virtual noon (HL condition), or with modulated UV radiation added (HL+UV condition), the UV dose at noon reaching 7.6 W m^-2 ^for UV-A and 0.6 W m^-2 ^for UV-B (see additional file 1: Fig. S1). Samples were then taken every hour during three consecutive days and the DNA content of cells was measured by flow cytometry (Fig. 1). In both light conditions, *Prochlorococcus *population growth conformed to the slow-growth case of Cooper and Helmstetter's prokaryotic cell cycle model [29], with only one DNA replication round per day. Indeed, as described before [6,7], *Prochlorococcus *DNA distributions always resembled the characteristic bimodal DNA distributions observed for eukaryotes, with a first discrete gap phase (G_1_), where cells possess one chromosome copy, preceding a well defined chromosome replication phase (S), followed by a second gap phase (G_2_), where cells have completed DNA replication but have not yet divided, and thus possess two chromosome copies (see additional file 2: Fig. S2). The G_1_/S/G_2 _designation will therefore be used in the text hereafter.

Figure 1 shows the time course variations of the percentages of cells in the different phases of the cell cycle. Under HL condition, cells started to enter the S phase about 4 h before the light-to-dark transition (LDT) and the peak of S cells was reached exactly at the LDT. The first G_2 _cells appeared at the LDT and the peak of G_2 _cells was reached 4 h later. Most cells had completed division before virtual sunrise, as shown by a percentage of cells in G_1 _close to 100% at (or 1 h after) that time (Fig. 1A). PCC9511 cultures acclimated to HL+UV conditions showed a remarkable cytological response with regard to the timing of chromosome replication. In the presence of UV, entry into S was clearly delayed, with the onset of chromosome replication occurring about 1 h before the LDT and the maximum number of cells in S phase reached 2 h after the LDT. Entry into G_2 _was also delayed by 3 h, but the peak of G_2 _cells was reached more quickly, so that it occurred on average only 1 h after that observed under the HL condition (Fig. 1B).

The faster progression of cells through S and G_2 _phases under HL+UV than HL only conditions in batch culture was confirmed by calculating the lengths of the S and G_2 _phases, which were shorter in the former condition (Table 1). Cells grown under HL+UV exhibited a higher level of synchronization (as shown by a lower synchronization index, S_r_) than those grown under HL only. However, the calculated growth rates were not significantly different between the two conditions. Therefore, the dose of UV irradiation that was used in this experiment did not prevent cells from growing at near maximal rate despite the delay of entry in S phase (Table 1). It must be noted that growth rates calculated from the percentages of cells in S and G_2 _(μ_cc_) using the method described by Carpenter & Chang [30] were systematically about 10% higher than those calculated from the change in cell number (μ_nb_). Since the latter method was used to assess the growth rate of continuous cultures (see below), these experiments in batch cultures were therefore useful to estimate the bias brought by these cell cycle-based growth rate measurements.

**Table 1:** Growth parameters of batch and continuous cultures of *Prochlorococcus marinus *PCC9511 grown under a 12 h/12 h light/dark cycle under HL supplemented or not with UV radiations.

	Batch Cultures		Continuous Cultures	
		
Growth parameters*	HL	HL+UV	HL	HL+UV
μ_cc _(d^-1^)	0.67 ± 0.05	0.68 ± 0.03	0.69 ± 0.09	0.66 ± 0.04
μ_nb _(d^-1^)	0.60 ± 0.13	0.62 ± 0.11	n.a.	n.a.
T_G1 _(h)	16.8 ± 1.6	18.4 ± 0.8	17.8 ± 2.5	19.0 ± 1.5
T_S _(h)	4.03 ± 0.30	3.47 ± 0.28	3.71 ± 0.77	3.83 ± 0.49
T_G2 _(h)	3.97 ± 0.30	2.53 ± 0.28	2.95 ± 0.31	2.51 ± 0.60
S_r_	32.4 ± 2.2	24.6 ± 1.1	27.2 ± 1.2	25.0 ± 1.4

Values are averages (± SD) of three consecutive days and two biological replicates

* Growth rates per day calculated from: cell cycle data (μ_cc_) or cell numbers (μ_nb_); T_G1_, T_S_, T_G2_: cell cycle phase duration in hours; S_r_: rate of synchronization estimated from the ratio (T_S_+T_G2_)/(T_G1_+T_S_+T_G2_)

n.a.: not applicable

### *Cell cycle dynamics of *P. marinus *PCC9511 cells in batch culture during shifts to a different light condition*

A second series of preliminary experiments in batch culture was performed to see i) whether changes in PAR level from modulated low light (LL; corresponding to a maximum irradiance level E_max _at noon ~ 100 μmol photons m^-2 ^s^-1^) to modulated HL (E_max _at noon ~ 900 μmol photons m^-2 ^s^-1^) would also affect the timing of the initiation of DNA replication in *P. marinus *cells and ii) how fast was the delay in chromosome replication observed when PCC9511 cells pre-acclimated to HL were suddenly exposed to HL+UV conditions.

**Figure 2:**
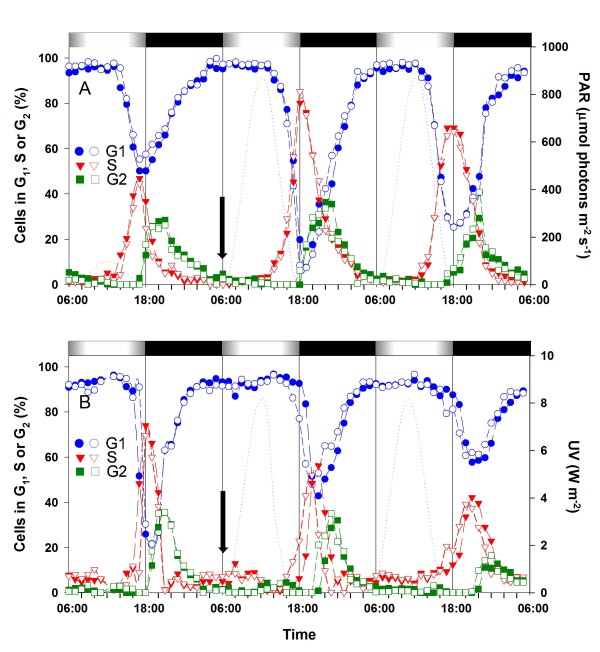
**Effect of shifting light/dark-entrained cultures to a new light condition on the cell cycle phase patterns of *Prochlorococcus marinus *PCC9511**. **A**, distribution of cells in G_1 _(blue), S (red) and G_2 _(green) phases for small volume batch cultures of PCC9511 acclimated under LL and shifted to HL conditions. **B**, HL acclimated cultures were followed during one L/D cycle then shifted to HL+UV conditions. The experiment was done in duplicates shown by filled and empty symbols. Note that only the UV radiation curve is shown in graph B since the visible light curve is the same as in graph A. Black arrows indicate the time point of the shift. White and black bars indicate light and dark periods. The dashed line indicates the growth irradiance curve (right axis). Abbreviations as in Fig. 1.

When acclimated to modulated LL, *P. marinus *cells generally started chromosome replication slightly earlier (LDT minus 5 h) than under HL conditions and the S phase maximum was also reached 1 h earlier (Fig. 2A). When shifted to HL, cells initiated DNA replication at the same time as in LL, but the peak of S cells was shifted to the LDT, as observed for HL acclimated cells. This event was accompanied by a notable increase in the peak height of the S cell maximum (from 48 to 85%) on the first day of increased PAR, but on the second day after HL shift, this percentage decreased to levels (ca. 65%) comparable to those observed in HL acclimated cultures (compare Figs. 1A and 2A). Indeed, PCC9511 cells grew much faster under HL than LL conditions and the maximal growth rate (comparable to that of HL acclimated cells) was reached already on the first day of increased PAR (Table 2). This enhanced growth rate resulted from a dramatic shortening of the G_1 _phase and, to a less extent, of the G_2 _phase, whereas the S phase was extended (Table 2). However, this rather long S phase, as compared to HL acclimated cells, suggests that cultures were not physiologically fully acclimated to the new light conditions, even two days after the shift.

**Table 2:** Growth parameters of PCC9511 batch cultures shifted from LL to HL during 12 h/12 h L/D cycles.

Growth Parameters*	Cycle 1 (LL)	Cycle 2 (HL)	Cycle 3 (HL)
μ_cc _(d^-1^)	0.43 ± 0.03	0.67 ± 0.01	0.62 ± 0.01
μ_nb _(d^-1^)	0.37 ± 0.04	0.59 ± 0.09	0.58 ± 0.05
T_G1 _(h)	30.8 ± 3.1	16.7 ± 0.3	18.8 ± 0.2
T_S _(h)	4.12 ± 0.01	5.15 ± 0.14	5.53 ± 0.12
T_G2 _(h)	3.89 ± 0.01	2.85 ± 0.14	2.47 ± 0.12
S_r_	20.8 ± 1.7	32.4 ± 0.4	29.8 ± 0.3

Values shown are averages (± mean deviation) of two biological replicates

* Growth rates per day calculated from: cell cycle data (μ_cc_) or cell numbers (μ_nb_); T_G1_, T_S_, T_G2_: cell cycle phase duration in hours; S_r_: rate of synchronization estimated from the ratio (T_S_+T_G2_)/(T_G1_+T_S_+T_G2_)

In the second shift experiment, HL acclimated PCC9511 cultures were sampled during one complete L/D cycle, then on the following two days were subjected to a modulated L/D cycle of HL+UV radiations. As for the HL+UV acclimated cells, UV exposure seemed to cause a delay in the initiation of DNA replication, but with the peak of S cells occurring 3 to 4 h after the LDT (Fig. 2B), instead of 2 h. Furthermore, although the UV dose received by the cells was the same in the UV acclimation and UV shift experiments, UV irradiation was clearly much more stressful for the cells in the second case, as they reacted by dramatically decreasing their growth rate (Table 3), an effect which was even more marked on the second day after switching the UV lamps on.

**Table 3:** Growth parameters of PCC9511 batch cultures shifted from HL to HL+UV during 12 h/12 h L/D cycles.

Growth Parameters*	Cycle 1 (HL)	Cycle 2 (HL+UV)	Cycle 3 (HL+UV)
μ_cc _(d^-1^)	0.69 ± 0.02	0.61 ± 0.01	0.45 ± 0.00
μ_nb _(d^-1^)	0.64 ± 0.05	0.45 ± 0.02	0.1 ± 0.02
T_G1 _(h)	18.0 ± 0.6	21.4 ± 0.3	29.3 ± 0.2
T_S _(h)	3.67 ± 0.14	3.72 ± 0.09	6.25 ± 0.03
T_G2 _(h)	2.33 ± 0.14	2.28 ± 0.09	1.75 ± 0.03
S_r_	25.0 ± 0.7	21.9 ± 0.2	21.5 ± 0.1

Values shown are averages (± mean deviation) of two biological replicates

*Growth rates per day calculated from: cell cycle data (μ_cc_) or cell numbers (μ_nb_); T_G1_, T_S_, T_G2_: cell cycle phase duration in hours; S_r_: rate of synchronization estimated from the ratio (T_S_+T_G2_)/(T_G1_+T_S_+T_G2_)

### *Comparative cell cycle dynamics of acclimated *P. marinus *PCC9511 cells grown in continuous cultures with and without UV radiation*

**Figure 3:**
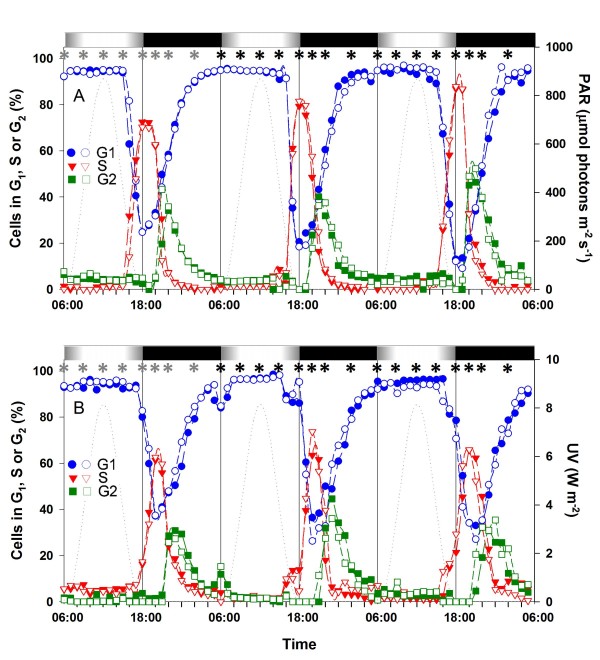
**Effect of UV exposure on the timing of the cell cycle phases of *Prochlorococcus marinus *PCC9511 cells grown in large volume, continuous cultures used for real time quantitative PCR (qPCR) and microarray analyses**. **A**, distribution of G_1 _(blue), S (red) and G_2 _(green) phases for large volume continuous cultures of PCC9511 grown acclimated to HL. **B**, same for HL+UV conditions. The experiment was done in duplicates shown by filled and empty symbols. Note that only the UV radiation curve is shown in graph B since the visible light (PAR) curve is the same as in graph A. Asterisks indicate the time points of sampling for qPCR (grey) and microarrays (black). White and black bars indicate light and dark periods. The dashed line indicates the growth irradiance (right axis). Abbreviations as in Fig. 1.

Large volume, continuous cultures of *P. marinus *cells acclimated to either HL or HL+UV conditions were used for gene expression analyses. These cultures were sampled for RNA eight times per day during three consecutive days. A shorter sampling interval (every 2 h) was used during the DNA replication period (Fig. 3), to closely analyze transcriptome changes caused by UV radiation during this critical phase of the cell cycle. The pattern of G_1_, S and G_2 _phases in HL+UV was similar to that in the batch experiments, with the same 2 h delay of the S phase into the dark period (Fig. 1). However, in HL conditions, the G_2 _maximum in continuous culture occurred on average 1 h earlier than in batch cultures due to a shorter G_2 _period and a better synchronization index of the whole population (Table 1). This is possibly linked to the particularly fast growth rate (μ_cc _of 0.71 d^-1^, corresponding approximately to a μ_nb _of 0.64 d^-1^) observed in this experiment (Table 1). Another notable difference between the two sets of experiments is the fact that during the second and third day in the continuous HL+UV culture, there was a shoulder on the left of the S peak (Fig. 3), suggesting that a small percentage of cells already had entered into S phase 2 h before the LDT, though the bulk of the cell population replicated DNA only during the dark period. The comparison of μ_cc _between batch and continuous cultures clearly demonstrated that the latter were growing exponentially in both HL and HL+UV conditions during the whole sampling period used for gene expression analyses.

### Effects of ultraviolet radiation on the whole transcriptome dynamics

Microarray analyses were used to identify which genes were differentially expressed between HL and HL+UV during the active phases of the cell cycle of *P. marinus *PCC9511, with the goal to understand the molecular bases of the delay of DNA replication in the latter condition. We made pairwise comparisons of microarray datasets corresponding to the same time points around the LDT in HL+UV and HL conditions, i.e. 15:00 (UV15 *vs*. HL15; corresponding to the G_1 _phase in each condition), 18:00 (UV18 *vs*. HL18), 20:00 (UV20 *vs*. HL20) and 22:00 (UV22 *vs*. HL22; corresponding to the G_2 _phase in each condition). To better analyze the changes in gene expression patterns occurring during the DNA synthesis (S) phase, we also compared samples taken at 20:00 in HL+UV and at 18:00 in HL (UV20 *vs*. HL18), respectively, as this corresponds to the maximum percentage of cells in S for each condition (see Fig. 3).

**Figure 4:**
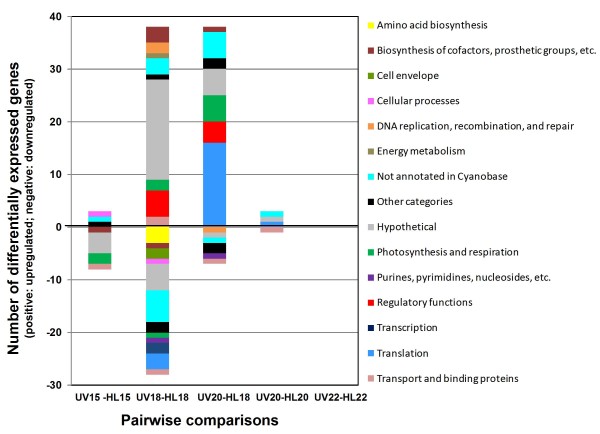
**Functional categories of the differentially regulated genes for the different pairwise timepoint comparisons**. LIMMA and Student's t-test were used to perform pairwise comparisons of different samples (UV15 *vs*. HL15, UV18 *vs*. HL18, UV20 *vs*. HL20, UV22 *vs*. HL22, UV20 *vs*. HL18) and genes with a log_2_(FC) > 1 and an adjusted p-value (FDR ≤ 0.1) with either one of these methods were selected to draw the bar chart.

Overall, 217 genes of the 1,963 analyzed genes (11.1%) showed statistically significant differential expression levels in all comparisons performed between the two light conditions, with a false discovery rate (FDR) ≤ 0.1 using t-test and/or LIMMA analyses (including 115 genes with significant fold change (FC) values, i.e. with log_2_(FC) > 1; see Fig. 4 and additional file 3: Table T1). The greatest number of differentially expressed genes was obtained for the UV18 *vs*. HL18 (136 genes, including 66 with log_2_(FC) > 1; Fig. 4) and the UV20 *vs*. HL18 comparisons (86 genes, including 45 with log_2_(FC) > 1; Fig. 4).

**Figure 5:**
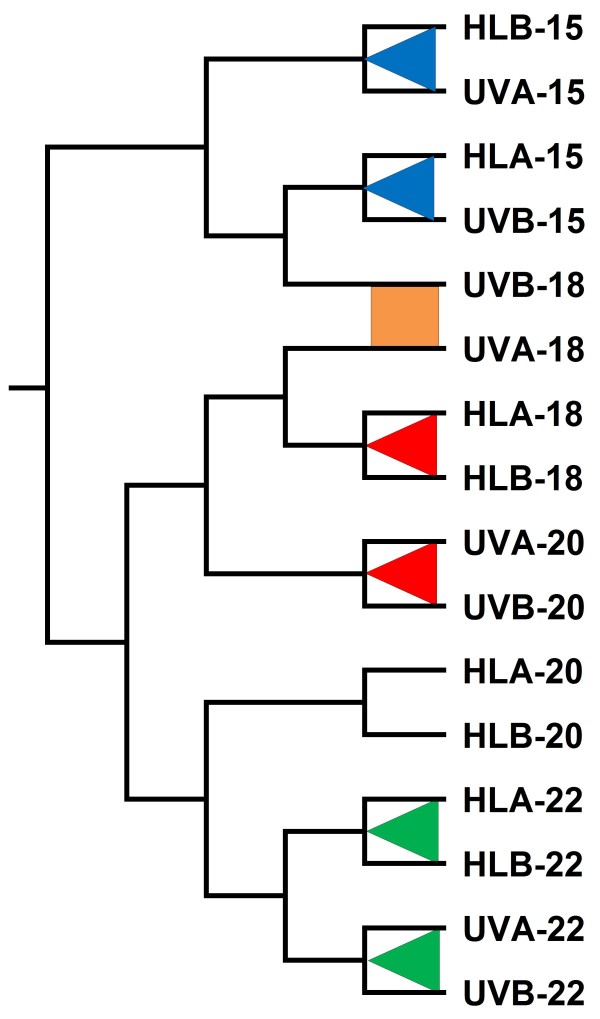
**Hierarchical clustering analysis of the microarray dataset**. Clustering analysis was performed on a selected gene list (819 genes) generated by one-way ANOVA with an adjusted p-value (FDR ≤ 0.1) and after combining data from days 1 and 2 for both cultures (A and B) and light conditions (HL and HL+UV) and at each time point. The dendrogram was produced as described in the text. Colored triangles correspond to the different cell cycle phases with G_1 _in blue, S in red and G_2 _in green. The orange square indicates the stage where cells exhibit a delay in the S phase under HL+UV condition.

Hierarchical clustering analysis using Pearson's correlation of the whole expression dataset (averaged over 2 consecutive days) showed that for any given light treatment and time of the day, cultures A and B grouped well together (Fig. 5). This showed that experimental conditions influenced the expression data more than did technical and biological variability between replicates. Furthermore, whole transcriptomic profiles clustered according to the sampling time and/or cell cycle stage, since UV15 and HL15 corresponded to G_1_, UV20 and HL18 to S, and UV22 and HL22 to G_2_. It is noteworthy that the two replicates of UV18 were not congruent, since sample B clustered close to HL15 and UV15, as expected for cells that are seemingly arrested in G_1_, whereas sample A clustered with the HL18 dataset, i.e. according to sampling time. Finally, the HL20 dataset clustered with the UV22 and HL22 datasets, consistent with the fact that part of the population of the HL20 sample was already in G_2 _(see Fig. 3A). Thus, it seems that the S phase delay had a strong effect on the PCC9511 transcriptome, competing with the strong effect of diurnal rhythm, since most genes are light-regulated in these organisms [14].

Among the statistically significant genes mentioned above, only 115 genes (or 53.0%) displayed fold changes higher than two-fold in HL *vs*. HL+UV timepoint pairwise comparisons (see Fig. 4 and additional file 3: Table T1). The following paragraphs discuss the most meaningful comparisons.

Eleven genes from this dataset were differentially expressed in UV15 *vs*. HL15 (G_1 _phase) and may be involved in the cell response to UV exposure. Seven of them were upregulated under HL+UV (see additional file 3: Table T1). These were one non-coding RNA (ncRNA, Yfr7; [28]), five photosynthetic genes, including *PMM1118*, one member of the high light inducible (*hli*) gene family (*hli04*), and *PMM0743*, an ortholog of *slr0228*, which encodes FtsH, a protein involved in D1 repair and degradation in *Synechocystis *sp. PCC6803 [31]. Consistently with quantitative PCR analyses (see below), the *PMM1697 *gene encoding the type II σ factor RpoD4 was downregulated at 15:00 in cultures exposed to HL+UV, though its p-value was statistically significant only before Benjamini and Hochberg (BH) adjustment (FDR ≤ 0.1; see additional file 3: Table T1).

The UV18 *vs*. HL18 comparison showed the largest number (66) of differentially expressed genes, as expected from the fact that cells were essentially in G_1 _in the HL+UV condition, whereas in HL most cells were in S (Fig. 3). One third of these genes (24) had no assigned function. The gene coding for one of the main subunits of the ATP synthase (*atpA*; *PMM1451*) was downregulated under HL+UV and most genes coding for other subunits of this complex (*atpD, E, F, G and H*, encoded by *PMM1452*, *PMM1439 *and *PMM1453-1455*, respectively) were also very close to the statistically significant fold change (FC) cutoff (see additional file 3: Table T1). If these relative reductions in the transcript levels of *atp *genes at 18:00 in the cells grown in HL+UV actually translated into a lower amount of ATPase produced, this could have resulted into a relative decrease (or delay) in energy supply of these cells during the dark period. Two key genes for the synthesis of RNA polymerase, i.e. *rpoA *(*PMM1535*), encoding the α subunit, and *PMM0496*, encoding the major σ factor RpoD1/SigA, were also expressed at much lower levels under HL+UV than HL conditions at 18:00. Assuming that this reduction resulted in correspondly lower protein levels, it is possible that the overall transcriptional activity of UV-acclimated cells could be reduced after the LDT. Since *PMM1629*, encoding the type II σ factor RpoD8, was upregulated under HL+UV, it is possible that RpoD8 replaces RpoD1 in the early dark period. The transcriptional regulator gene *pedR *(*PMM0154*) and two genes potentially involved in DNA repair (*PMM1528 *and *PMM0843*, encoding respectively an HNH endonuclease and a possible TldD-like modulator of DNA gyrase) were also upregulated at 18:00 in the HL+UV condition (see additional file 3: Table T1), suggesting that the latter genes were directly or indirectly involved in the repair of DNA damage caused by UV irradiation.

Surprisingly, the UV20 *vs*. HL18 comparison also revealed a high number of up- or downregulated genes (45), suggesting that although cells were predominantly in S phase in both light conditions, UV irradiation during the day altered differentially the pattern of expression of genes from the different metabolic pathways around the LDT. Among annotated genes of this dataset, those most represented belonged to the functional categories of ribosomal proteins (14, all upregulated under HL+UV; see Fig. 4 and additional file 3: Table T1). However, most of these genes were also upregulated in the HL20 *vs*. HL18 comparison (data not shown), indicating that the diel expression pattern of these key translation genes was less affected by UV stress than by daytime, at least around the LDT period. Most of the genes that were differentially regulated in the UV20 *vs*. HL18 but not in the HL20 *vs*. HL18 comparisons belonged to the conserved hypothetical gene category (data not shown).

Few genes were differentially expressed between HL and HL+UV during the dark period (4 genes in the UV20 *vs*. HL20 and none in the UV22 *vs*. HL22 comparisons, corresponding to the G_2 _phase and the beginning of cell division, respectively; Fig. 4) and most of them were not assignable to a characterized functional category (see Fig. 4 and additional file 3: Table T1). This suggests that the effect of UV irradiation on the PCC9511 transcriptome was no longer significant only a few hours after the LDT.

Altogether, surprisingly few genes belonging to pathways directly linked to the cell cycle crossed the statistical significance (FDR < 0.1) and FC [log_2_(FC) < -1 or > 1] cutoffs (see additional file 3: Table T1). To insure that this was not due to a lack of sensitivity of the arrays and to gain more detailed information on the behavior of this gene category, seventeen genes were selected and subsequently analyzed by real time quantitative PCR (hereafter qPCR). This set includes genes that were either differentially expressed in microarray analyses or representative of key processes, including DNA replication, cell division, DNA repair, transcriptional regulation and the circadian clock. All genes that exhibited significantly different expression levels (i.e., with FDR ≤ 0.1) in one of our comparisons in microarray analyses showed a similar response (up- or downregulation) in qPCR experiments [Pearson's correlation coefficient of 0.86 for pairwise comparisons with a log_2_(FC) < -0.5 or > 0.5].

### Expression patterns of genes involved in the initiation of chromosome replication and cell division are strongly affected by UV radiation

**Figure 6:**
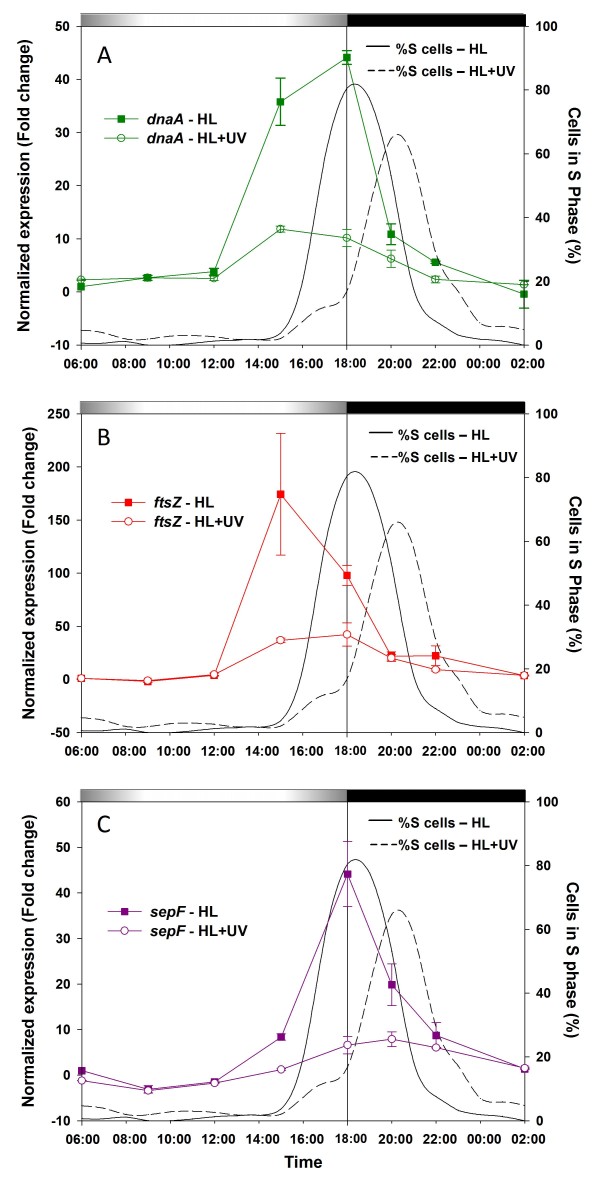
**Gene expression patterns of L/D-synchronized *Prochlorococcus marinus *PCC9511 cultures under HL and UV growth conditions, as measured by qPCR**. **A**, *dnaA*. **B**, *ftsZ*. **C**, *sepF*. The percentage of cells in the S phase of the cell cycle under HL (solid line) and HL+UV (dashed line) are also shown for comparison. Error bars indicate mean deviation for two biological replicates. For each graph, transcript levels were normalized to the reference time point 6:00 in HL condition. Grey and black bars indicate light and dark periods.

Three genes were selected as representatives of the DNA replication and cell division pathways, *dnaA *(*PMM0565*), encoding the DNA replication initiation protein DnaA, *ftsZ *(*PMM1309*), encoding the tubulin homolog GTPase protein FtsZ, which forms a ring-shaped septum at midcell during cell division, and *sepF *(*PMM0395*), encoding a protein involved in the assembly and stability of the FtsZ ring [32]. The transcript levels of all three genes exhibited strong temporal variations during the diel cycle in both light conditions (Fig. 6). Under HL+UV conditions, although expression levels of both *dnaA *and *ftsZ *genes significantly increased at 15:00 compared to the 6:00 time point, the expression level was 3- to 5-fold lower than under HL at 15:00. The *sepF *gene expression pattern was characterized by a strong peak at the LDT in HL, but like for the other two genes, the diel variations of *sepF *expression levels were dramatically reduced in UV-irradiated cells. In both light conditions, the *sepF *expression was maximum during the S phase (Fig. 6C).

### Transcript levels of DNA repair genes are moderately affected by UV radiation

**Figure 7:**
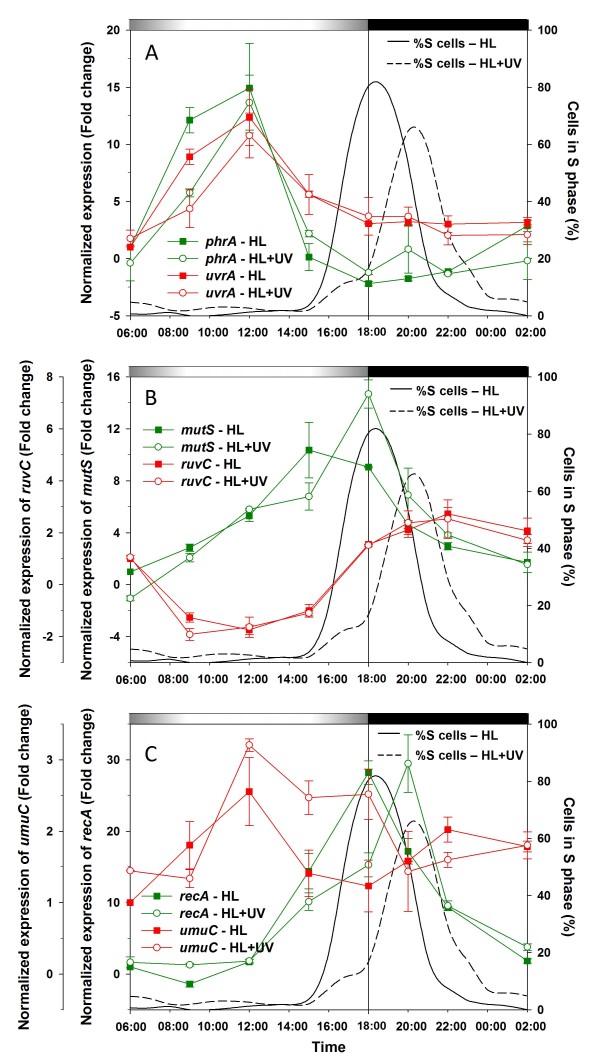
**Gene expression patterns of L/D-synchronized *Prochlorococcus marinus *PCC9511 cultures under HL and UV growth conditions, as measured by qPCR**. **A**, *phrA *and *uvrA*. **B**, *mutS *and *ruvC*. **C**, *recA *and *umuC*. The percentage of cells in the S phase of the cell cycle under HL (solid line) and HL+UV (dashed line) are also shown for comparison. Error bars indicate mean deviation for two biological replicates. For each graph, transcript levels were normalized to the reference time point 6:00 in HL condition. Grey and black bars indicate light and dark periods.

Analyses of diel expression patterns of six genes representative of different DNA repair pathways were compared between HL and HL+UV conditions (Fig. 7). These patterns were very different among the six genes, suggesting a refined orchestration of the different pathways. A first set of DNA repair genes, including *phrA *(*PMM0285*), which codes for a DNA photolyase and *uvrA *(*PMM1712*), encoding the subunit A of the excinuclease UvrABC, an enzyme of the nucleotide excision DNA repair (NER) pathway, was strongly expressed during the light period. Their expression levels followed more or less closely the diel cycle of irradiance (Fig. 7A). Interestingly, the relative expression levels of both genes were already high under HL and exposure to UV radiations did not provoke any further increase of these levels, even at midday. The only notable difference between the HL and HL+UV profiles was a slightly higher expression level at 9:00 am for both genes in the former condition (Fig. 7A).

Expression levels of *mutS *(*PMM1645*), a gene involved in the DNA mismatch repair (MMR) pathway, rose throughout the light period at the same rate in both light conditions, peaked right before the peak of S cells (i.e. 3 h before the LDT in HL and at the LDT in HL+UV), then decreased during the dark period (Fig. 7B). In sharp contrast with other DNA repair genes, the *ruvC *gene (*PMM1054*), which encodes the subunit C of the RuvABC resolvase endonuclease, an enzyme involved in recombinational DNA repair processes by homologous recombination, was downregulated during the daytime and was only induced at the LDT (Fig. 7B). It showed no response to the addition of UV radiation.

Among all DNA repair genes, the diel expression pattern of *recA *(*PMM1562*), which encodes an ATPase involved in repair of DNA double-strand breaks (DSBs) by homologous recombination, was seemingly the most affected by the presence of UV radiation. This pattern closely resembled that of *sepF*, with expression maxima concomitant with the S peak in both light conditions (i.e. delayed in HL+UV; Fig. 7C). However, in contrast to *sepF*, the height of the expression peak (normalized to the 6:00 level in HL) was similar between HL and HL+UV conditions (Fig. 7C). The temporal expression pattern of *umuC *(*PMM0937*), encoding a subunit of the error-prone polymerase V (PolV), was also somewhat affected by UV exposure, since in HL+UV, the gene remained highly expressed for 8 h after the midday maximum, whereas in HL only, *umuC *gene expression decreased sharply after the noon expression peak (Fig. 7C). This suggests that cells which were exposed to UV irradiation before entering S phase might use the DNA translesion synthesis (TLS) pathway [33] in order to overcome UV-induced lesions potentially blocking DNA replication.

### Global transcription regulators and circadian clock genes are mildly affected by UV stress

**Figure 8:**
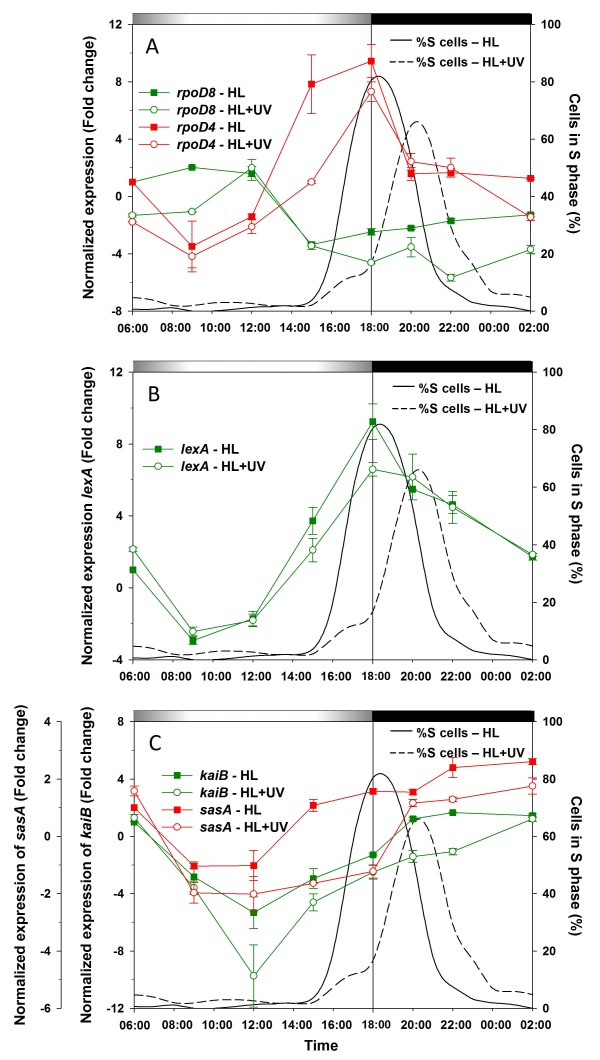
**Gene expression patterns of L/D-synchronized *Prochlorococcus marinus *PCC9511 cultures under HL and UV growth conditions, as measured by qPCR**. **A**, *rpoD8 *and *rpoD4*. **B**, *lexA*. **C**, *kaiB *and *sasA*. The percentage of cells in the S phase of the cell cycle under HL (solid line) and HL+UV (dashed line) are also shown for comparison. Error bars indicate mean deviation for two biological replicates. For each graph, transcript levels were normalized to the reference time point 6:00 in HL condition. Grey and black bars indicate light and dark periods.

RNA polymerase sigma factors are transcriptional regulators involved in the response of cyanobacteria to a variety of stress conditions [34]. The *Prochlorococcus marinus *PCC9511 genome encodes five sigma factors [4], which have been named here mainly following the nomenclature used for *Synechococcus *sp. PCC7942 [35] (see Cyanorak database: http://www.sb-roscoff.fr/Phyto/cyanorak/). This includes one member of the principal group 1 sigma factor (PMM0496, RpoD1), and four members of the group 2 sigma factors (PMM1697, RpoD4; PMM1289, RpoD6; PMM0577, RpoD7 and PMM1629, RpoD8), of which RpoD7-8 are specific for marine picocyanobacteria [34]. In the present study, we used a qPCR approach to examine the expression of *rpoD4 *and *rpoD8*, which were previously shown to have very distinct diel patterns under modulated diel cycles of PAR [14,36]. The *rpoD8 *gene was upregulated earlier in HL than HL+UV conditions, with equivalent expression at noon under both growth conditions, then downregulated during the rest of the day with a greater decrease throughout the subjective night period under HL+UV growth conditions (Fig. 8A). This pattern was completely the opposite of *rpoD4*, which was expressed at a low level until noon in HL (or until 15:00 in HL+UV), was strongly upregulated at the LDT, then returned to the same expression level as at 6:00 (or even less in HL+UV) for the rest of the dark period (Fig. 8A).

The *lexA *gene (*PMM1262*) encodes a transcriptional regulator, which in *Escherichia coli *governs the SOS DNA damage repair response [37]. Like *rpoD4*, the *lexA *RNA level was the lowest during the morning hours, then strongly increased after midday so that expression was maximal at the LDT and decreased slowly thereafter (Fig. 8B). The pattern was similar in both light conditions, except that the peak in HL+UV was slightly lower.

Two genes linked to the circadian clock machinery were also studied, *kaiB *(*PMM1343*), encoding one of the only two core clock proteins (since all *Prochlorococcus *strains lack KaiA [36]) and *sasA *(*PMM1077*), coding for a two-component sensory transduction histidine kinase which relays clock output signal to downstream genes [38]. In HL, the level of *kaiB *mRNA decreased during the first hours of the light period, reaching a minimum at noon and then increasing until 20:00, when it reached an expression level similar to the 6:00 reference level (Fig. 8C). In HL+UV, *kaiB *expression pattern was generally the same as in HL, except that its relative expression level was two-fold lower at noon, then increased progressively to reach the reference expression level at approximately 2:00. As already noted in a previous study [14], diel changes in *kaiC *gene (*PMM1342*) expression levels were very low, with no significant differences under HL and HL+UV growth conditions (data not shown).

A diel cycle in the transcript levels of the *sasA *gene was also observed. In HL, it roughly followed that of *kaiB *except that there was no mimima at noon, but rather a long period of downregulation lasting from 9:00 to 18:00, then a slight upregulation at the beginning of the night (Fig. 8C). In the presence of UV, the relative *sasA *expression levels were lower than in HL during most of the day, consistent with the effect of UV irradiation on *kaiB *RNA levels. The most notable difference between the two light conditions is (as for *ruvC*) that the switch from down- to upregulation of *sasA *was delayed in HL+UV and concomitant with the S peak (Fig. 8C), suggesting a possible involvement of circadian clock output signals on timing of cell cycle progression in PCC9511.

## Discussion

### Importance of the modulated character of UV radiation and of light history on the response of *Prochlorococcus *cells to UV stress

Several field studies using on-deck incubations have suggested that the marine cyanobacterium *Prochlorococcus *is particularly susceptible to the direct (or indirect, i.e. via the generation of reactive oxygen species) effects of UV irradiation, in particular in comparison to the co-occurring and phylogenetically closely related genus *Synechococcus*, which is seemingly much more resistant to UV stress [39,40]. This apparent sensitivity has been attributed in part to the tiny size of *Prochlorococcus *cells as well as their streamlined genomes, encompassing a minimal gene complement for a phototroph and hence reduced UV protection machinery [23,25,41]. Still, *Prochlorococcus *is very abundant in the upper layer of most oligotrophic waters (with the notable exception of the S Pacific gyre; see [3]) and can sustain high growth rates in near surface, UV-irradiated waters [7,8,42-44].

In order to better understand the molecular mechanisms by which *Prochlorococcus *manages to cope with UV stress, we grew *P. marinus *strain PCC9511 under quasi natural light conditions by using a custom-designed illumination system which provided a modulated L/D cycle of PAR and UV radiation. This system induced a very tight synchronization of cell cycle and division (Figs. 1 and 3). Most studies that have analyzed UV effects on cyanobacteria thus far have been performed on asynchronously growing cells either by abruptly subjecting cultures to short-term UV stress (see e.g. [45-47]) or longer term acclimation to constant UV exposure [48,49]. The long term (acclimation) response of cells is known to be significantly different from the short term (shock) response, as it involves different sets of genes and regulation networks [48]. Yet, the modulated character of UV stress in nature, its co-occurrence with high light stress (also modulated) and the existence of long, dark recovery periods (i.e., nights) are also very important factors to take into account to fully understand how cells can acclimate to UV stress in nature. The dynamic aspect of this stress triggers a succession of signalling, gene regulation and/or repair pathways that lead to a temporally complex, coordinated response [50]. This finely tuned orchestration of the transcriptome and metabolome cannot be observed after merely subjecting cultures to a continuous (and often harsh) UV treatment, as it generally provokes a "distress" response that may eventually activate programmed cell death [51-53]. In our experiments, even though *P. marinus *sp. PCC9511 was growing at similar rates (ca. 1 division per day) in HL and HL+UV conditions (Figs. 1 and 3; Table 1), this strain could not tolerate a sudden shift from HL to HL+UV conditions, as this provoked a sharp decrease of its growth rate (Fig. 2B and Table 3) and ultimately death of the culture within a few days (not shown). To successfully acclimate our cultures to our experimental HL+UV conditions, we therefore had to increase the UV dose incrementally with several days of acclimation at each step (see methods). Thus, acclimation of *Prochlorococcus *cells to UV stress is the result of a very subtle balance between the light environment experienced by cells in their specific niche (encompassing diel variations of visible and UV radiations) and a precise temporal succession of metabolic and repair processes that closely matches the ambient level of stress at any time of the day. Hence, attempts to sample cells from their natural environment and to incubate them in other (even slightly different) conditions, (as usually done to study the effects of UV stress *in situ *[39,40] might well disrupt this fragile balance and rapidly lead to cell death.

It must be stressed that i) this hypothesis does not necessarily apply to other cyanobacteria that have a larger variety of UV protection systems [53] or at least (in the case of marine *Synechococcus*) a larger set of DNA repair genes (e.g. several putative photolyases), conferring them with a better resistance to UV stress, and ii) PCC9511 seems to cope with high light much better than with UV shock, since after cultures were shifted from LL to HL, their growth rate increased to one doubling per day by the day after the shift (Table 2). In contrast, LL-adapted *Prochlorococcus *spp. strains (such as SS120 or MIT9313) seemingly need to be acclimated incrementally to higher irradiances [54].

### Molecular bases of the chromosome replication delay

One of the main results of the present study is that *P. marinus *PCC9511 can acclimate to relatively high doses of UV irradiation (commensurate with those that cells can experience in the upper mixed layer of oceans) by delaying DNA synthesis (S phase) towards the dark period. This strategy could reduce the risk of UV-induced replication errors [50]. It is probable that this delay is also needed for cells to repair UV-induced damages to DNA accumulated during the period preceding chromosome replication. In UV-irradiated cultures, we sometimes observed that a minor fraction of the population seemingly initiated chromosome replication at 15:00 (i.e. similar to the HL condition), as suggested by the shoulder to the left of the S peak before dusk (Fig. 3B). However, the absence of any skew on the left of the corresponding G_2 _peak suggests that these cells either had an extended S phase (i.e. were temporarily blocked in S) or died before completing DNA replication. The maintenance of a high growth rate under HL+UV conditions favors the former hypothesis.

Most UV-irradiated cells could not enter the S phase before complete darkness. One may wonder whether this observation is compatible with the occurrence of a UV stress-induced cell cycle "checkpoint", i.e. "a regulatory pathway that controls the order and timing of cell cycle transitions and ensure that critical events such as DNA replication and chromosome segregation are completed with high fidelity" [55]. If it exists, this checkpoint could be a "DNA replication initiation checkpoint", i.e. located before the G_1_-S transition. However, this hypothesis would not account for the previously mentioned small percentage of the population that was seemingly blocked in S. The occurrence of a "DNA replication completion checkpoint" was suggested for UV-C irradiated *E. coli *cells [56]. Cells in G_1 _could not start chromosome replication while S cells could not complete replication and hence divide; only cells already in G_2 _at the time of irradiation were able to complete cytokinesis. In our case, however, because of the tight synchronization of the population, virtually no cell was sufficiently advanced in the cell cycle during the pre-dusk period to complete cytokinesis.

It is generally thought that checkpoints are controlled by specific protein complexes involved in signaling (photoreceptors) and/or checking [57]. Thus, *Prochlorococcus *might possess a UV sensor which, when detecting these wavelengths, could launch a cascade of controlling mechanisms ultimately stopping the replication machinery. A UV-B sensor was characterized in the diazotrophic cyanobacterium *Chlorogloeopsis *sp. PCC6912 and was shown to mediate the induction of mycosporine-like amino acids synthesis [58]. However, no evidence for such a UV sensor is available in *Prochlorococcus *and, as argued later in this paper, its presence is rather unlikely. Recently, Cooper [59] proposed that checkpoints may in fact result from purely internal controls. It is possible that PCC9511 cells actually entered the early S phase but that the extensive occurrence of replication fork stalling due to accumulated DNA lesions and the elevated need for recovery of the replication process by lesion removal and replisome reloading [60] slowed down or even arrested the whole DNA synthesis process for a few hours, therefore explaining the observed delay without any need for a light sensing signal. The fact that UV-acclimated cultures did not show any obvious decrease in their overall growth rate indicates that if stalling of replication forks occurred, efficient DNA repair mechanisms must have allowed those cells blocked in S to restart and complete chromosome replication.

### UV stress leads to the downregulation of DNA replication and cell division genes

To further our understanding of the molecular bases of the observed delay in S phase completion, we analyzed the expression of key genes involved in chromosome replication and cell division. As is typically observed in model bacteria [61,62], the *dnaA *gene, encoding the master initiator protein of chromosome replication, was induced just before entry of cells into the S phase. Although an increase in *dnaA *expression occurred at the same time under HL and HL+UV, its level of expression was considerably lower in the latter condition. It is well known in *Escherichia coli *that initiation of chromosome replication depends on reaching a threshold level of DnaA protein [63]. Thus, it is plausible that the low amounts of *dnaA *transcripts at 15:00 in UV-irradiated PCC9511 cells (as compared to those in HL) may have resulted in a decreased rate of DnaA protein accumulation in the cell, resulting in a several hour delay in the time at which the DnaA threshold concentration is reached. No homologs of regulators (e.g. *seqA*, *dam, hda*) known in other bacteria to control the mode of action of DnaA [64] have yet been identified in PCC9511. Still, one possible regulatory mechanism may involve ATP, since it is a necessary co-factor transforming the inactive form of DnaA (DnaA-ADP) into its active form (DnaA-ATP), capable of initiating chromosome replication [65]. We hypothesize that the lowered expression levels of ATP synthase genes in HL+UV during the daytime, as seen both in microarray (for *atpA, D, E, F, G and H*; see above) and qPCR analyses (for *atpD *and *atpH*; see additional file 4: Fig. S3) could have caused a decrease in intracellular ATP levels that might have also contributed to delayed DnaA induction activity in PCC9511.

Even if the lowered expression of *dnaA *is sufficient by itself to explain the observed S phase delay, it appears that UV exposure also strongly affected the expression of several (and possibly all) genes involved in cell division, including *ftsZ *and *sepF*, both encoding key components of the divisome [66]. This similar behavior suggests that the DNA replication and cell division machineries could be controlled by the same regulatory network, though the timing of maximal expression varies between genes (Fig. 6). SepF is thought to be involved in the polymerization and stability of FtsZ filaments. Marbouty and co-workers [32] showed *in vitro *that SepF binds to preassembled FtsZ polymers, suggesting that SepF is required only after all the FtsZ protofilaments needed to make a Z-ring have been synthesized. This hypothesis is consistent with the delay observed between the peaks of expression of *ftsZ *and *sepF *in both light conditions.

### DNA repair genes are activated under high light

Another surprising result from this study is that UV exposure did not result in any significant upregulation of DNA repair genes (relative to HL conditions), including some which are known to be involved in repairing damage specifically induced by UV stress. This includes the *phrA *gene, which encodes an enzyme involved in repair (by photoreactivation) of the most frequent DNA lesions in response to UV, i.e. cyclobutane pyrimidine dimers (CPDs; [67]). Our results demonstrate that *phrA *is also strongly expressed under HL, with a pattern during the day that somewhat matched the irradiance curve, suggesting that the expression of this gene is strongly regulated by light. Recently, Osburne and co-workers [68] described a mutant of *P. marinus *MED4 exhibiting high resistance to UV stress. By comparing the whole genomes of the mutant and wild type, they could only find a single point mutation, located upstream of a two-gene operon consisting of *phrA *(called "*phrB*") and a gene coding for a nudix hydrolase (annotated "MutT", though its specific substrate is not known). This mutation resulted in the constitutive expression of this operon even under non-inductive conditions, suggesting that the occurrence of high levels of DNA photolyase and nudix hydrolase in the cells prior to UV treatment conferred these cells with better resistance to this stress than wild type cells, which needed some time to synthesize those proteins. In order to exclude the possibility that the PCC9511 strain used in our experiments possessed the point mutation described by Osburne and co-workers [68], we used the PCR primers defined by authors to amplify this region directly from cells collected from each duplicate culture of the HL and HL+UV experiments. In all cases, the sequences were the same as for the wild type (L. Garzarek and M. Ratin, unpublished data). It is noteworthy that Zinser and co-workers [14], who studied the diel variations of the whole transcriptome of L/D synchronized MED4 cultures, observed a very different expression pattern for *phrA *as we did here (Fig. 7A), with an increase at night and a decrease during the day (see [69]). Since they used a moderate light irradiance, reaching only one fourth of our HL conditions at virtual noon (232 *vs*. 875 μmol photons m^-2 ^s^-1 ^in the present study), it is possible that high PAR conditions are needed to trigger the synthesis of the DNA photolyase.

The *uvrA *gene showed an expression pattern very similar to that of *phrA *in both conditions. It encodes the DNA damage recognition component of the UvrABC system which in bacteria and archaea is involved in the nucleotide excision repair pathway (NER) [70]. This pathway, which has the ability to repair a wide range of structurally unrelated DNA lesions [71], is seemingly fully functional in *P. marinus *PCC9511, since it possesses conserved homologs of all three subunits of the UvrABC system. In Zinser and coworkers' study [14], *uvrA *transcript levels showed a rapid increase at the beginning of the light period, remained at quasi steady state during the rest of the day, then decreased at night (see [69]). This indicates that the *uvrA *system is also activated at moderate light, though it might not need to be adjusted as precisely to the ambient light as under HL.

Another essential safeguard of genomic integrity in prokaryotes is the DNA mismatch repair (MMR) pathway, which removes base mispairings, unpaired bases, and small insertion or deletion loops in DNA by the concerted action of MutS-L-H repair proteins [72]. The genome of *P. marinus *MED4 contains one homolog of *mutS*, which in *E. coli *encodes the DNA damage recognition component of the MMR system. Transcript levels of *mutS *were the lowest at dawn, increased continuously during the light period and decreased at the beginning of the S phase, suggesting that expression of this gene could increase together with the accumulation of UV and/or reactive oxygen species-induced mutations to DNA. However, no homologs of *mutL*, encoding an ATPase that forms a complex with MutS once the latter has recognized a DNA lesion, and *mutH*, coding for a restriction endonuclease that cleaves DNA at GATC sites, can be found in the MED4 genome (and hence PCC9511). The following step of the MMR process, i.e. DNA excision, is ensured in *E. coli *by several genes, including *recJ*, which encodes a single-stranded DNA-specific exonuclease and the *xseAB *operon, which encodes the two subunits of the exodeoxyribonuclease VII [72]. Surprisingly, homologs of these genes can be found in the genomes of the low light-adapted *Prochlorococcus *ecotypes, but not in high light adapted ecotypes, including MED4 [3]. Thus, even though putative homologs of enzymes involved in DNA resynthesis (the last step of MMR [72]) are present in MED4, including SSB, which has been implicated in the repair of single strand breaks, and several DNA ligases (in addition to the universal, error-free replicative DNA polymerase III, or Pol III, which is also involved in this process), biochemical studies are needed to determine whether MutS is associated with an MMR-like system in HL-adapted *P. marinus *strains or if this system is absent in these organisms.

Expression patterns of the *umuC *gene, encoding the subunit C of the UmuD'_2_C error-prone DNA polymerase V (Pol V), indicate that DNA translesion synthesis (TLS) reactions, used to bypass lesions in DNA templates on which Pol III usually stalls, occur in PCC9511 [73]. The *umuC *gene expression increased during the G_1 _phase with a peak at noon and was downregulated during the S phase. Interestingly, in HL+UV conditions, its expression level remained high during the entire period of S blockage. Posttranslational activation of Pol V requires the presence of RecA nucleoprotein filaments bound to ssDNA in order to generate its catalytically active form [74]. One can therefore speculate that, even though *umuC *expression was upregulated in the middle of the day under HL+UV conditions, the transcriptional repression of *recA *during that time may have delayed activation of Pol V. As a result, stalled replication forks may have taken longer to be rescued [75], providing another possible cause for the delay in S maximum observed under HL+UV. The *umuCD*-dependent cell cycle checkpoint model proposed for *E. coli *[57] may thus be applicable to *P. marinus *PCC9511.

While the NER (and possibly MMR) pathway is mainly active during the G_1 _phase, *Prochlorococcus *cells seem to activate another DNA repair system after the initiation of chromosome replication, namely the homologous recombination pathway that acts on double strand breaks. In this process, RuvA and RuvB, form a complex that promotes branch migration of Holliday junctions, then the endonuclease RuvC resolves the Holliday junctions by introducing nicks into DNA strands [76]. The fact that the diel expression pattern of the *ruvC *gene was similar under HL or HL+UV conditions suggests that the homologous recombination pathway is likely independent of the transcriptional control by the LexA/RecA system (see below), as is also the case in the freshwater cyanobacterium *Anabaena *sp. PCC7120 [77].

### Transcriptional regulation of the SOS response by LexA

The LexA protein of *E. coli *is a transcriptional repressor of the SOS DNA damage repair response, which is induced upon recognition of DNA damage caused by a wide range of intra- and extracellular elicitors, including UV-irradiation, oxidative stress and DNA replication abnormalities [78]. In PCC9511, the *lexA *expression pattern was almost the same under HL and HL+UV, suggesting that it is oxidative stress rather than UV which is the inducing factor for *lexA *expression. At a molecular level, de-repression of the forty-three genes constituting the *lexA *regulon in *E. coli *[79] is dependent upon the autocatalytic cleavage of the LexA protein, which is stimulated in response to DNA damage by interaction with ssDNA-RecA filaments [37]. This repressor cleavage reaction in *E. coli *requires several conserved sequence motifs in the LexA repressor, a catalytic serine nucleophile (S119), a basic lysine residue (K156) and an alanine-glycine cleavage bond (A84-G85) [80]. Absence of the LexA nucleophile and cleavage bond, a lack of *lexA *DNA damage inducibility in *Synechocystis *sp. PCC6803 [81] and its involvement in carbon fixation led Domain and co-workers [82] to question whether the *E. coli *type SOS regulon was conserved in cyanobacteria. However, sequence analysis of the LexA protein encoded by *P. marinus *MED4 shows that these three sequence motifs are conserved (see additional file 5: Fig. S4). Furthermore, a search for the LexA binding site in several *Prochlorococcus *genomes, including MED4 [83], uncovered the consensus motif TAGTACA-N_2_-TGTACTA upstream of the *recA*, *umuC *and *umuD *genes as well as *lexA *itself, a motif which is similar to the previously described consensus LexA site of gram-positive bacteria [77]. Therefore, unlike *Synechocystis *sp. PCC6803, it seems that *P. marinus *PCC9511 could well possess a LexA-regulated DNA repair system similar to that in *E. coli*. The different expression patterns of the LexA-controlled genes might reflect differences in the sequence conservation of this motif relative to the LexA consensus sequence [84]. Still, the late occurrence during the cell cycle of the *lexA *gene expression peak and its concomitance with the *recA *expression maximum in HL conditions is somewhat surprising, given that their products act as repressor and activator of the SOS response, respectively [78] and one might have expected some differential expression patterns. The delay of the *recA *but not *lexA *expression peaks in UV-irradiated cells is therefore worth noting in this context as it is more compatible with the expected succession of LexA and RecA regulators in the frame of a typical, coordinated SOS response to DNA damages [37].

### Effect of UV on sigma factors and clock gene expression

Zinser and coworkers [14] recently showed that the five sigma factors of *P. marinus *MED4 were differentially regulated by light and suggested that this differential phasing, which is in agreement with the idea that they compete for the same core RNA polymerase, contribute to the variety of diel gene expression patterns observed within the whole transcriptome. In order to gain insight into the effects of UV irradiation on the diel RNA accumulation patterns of these expression regulators in PCC9511, we studied the expression of two group II sigma factors *(rpoD4 *and *rpoD8*). Their patterns of expression, which are globally consistent with those reported earlier [14,36], suggests that *rpoD8 *is maximally expressed shortly after dawn and one can hypothesize that its gene product (RpoD8) could control the expression of genes upregulated in the morning (such as *phrA*, *uvrA *and *umuC*). Similarly, *rpoD4 *RNA levels peak at LDT, and it is possible that RpoD4 could control the expression of genes expressed during this period (such as *recA*, *sepF *and *lexA*). The presence of UV radiation appeared to affect the expression patterns of both sigma factor genes. For *rpoD8*, because the daily amplitudes of variation were relatively modest (given that FC values ranging between -1 and +1 meant that genes were not differentially expressed; see methods), the differences observed during the light period might not be significant. In contrast, for *rpoD4*, there was a clear decrease in its relative expression at 15:00 in HL+UV compared to HL conditions, which could potentially result in a delay in the expression of the whole set of genes under the control of this sigma factor.

It has been proposed that the RpoD2 sigma factor of *Synechococcus *sp. strain PCC7942 is involved in a circadian clock output pathway [85]. There is no direct ortholog of of the *rpoD2 *gene in MED4 (and hence PCC9511), but one or several of the five sigma factors of this strain might have a similar function. The observed down-regulation of the circadian clock core oscillator *kaiB *gene at noon under HL+UV conditions could result in a modification of the diel expression patterns of one or several of these sigma factors, which in turn modified the expression of genes under their control (see above). Another gene known to convey the circadian clock output signal is *sasA*, which encodes a sensory histidine kinase. Like *kaiB*, it is maximally expressed during the night and its expression dramatically decreased at the beginning of the light period. However, while in HL it recovered its expression just after noon, this recovery took much longer in the presence of UV radiation, which could also potentially affect expression of the whole transcriptome. Indeed, SasA plays a key role in chromosome condensation and superhelicity status, which are known to regulate global gene expression and separation of replicated chromosomes [86].

## Conclusion

In this study, we analyzed the response of *Prochlorococcus marinus *PCC9511 to an environmentally relevant UV stress, provided as a modulated light/dark cycle, as occurs in nature. Our results show that the primary response of UV-irradiated *Prochlorococcus *cultures involves a shift of chromosome replication phase towards the dark period, potentially minimizing the risk of UV-induced replication errors. Since the genes involved in DNA replication and cell division are most affected by UV stress, this delay of the S phase is probably related to the strong repression of those genes, in particular *dnaA*.

Another important outcome of this work is that the strong synchronization of the PCC9511 cells entrained by the modulated light-dark cycle allowed us to observe a clear temporal succession of the expression of genes encoding components of the different DNA repair pathways through the day. The first line of defense is provided by the light-dependent repair of CPDs by the DNA photolyase and removal of damaged oligonucleotides by NER. The presence of a light-regulated *mutS *gene suggests a possible involvement of MMR during G_1_, but we have no clear evidence yet that a fully operational MMR system exists in PCC9511. At later stages of the L/D cycle, when irradiation levels reached their maxima, *recA *and *lexA *expression increase. We hypothesize that the SOS response of PCC9511 is activated later in the afternoon due to LexA inactivation, resulting in the de-repression of genes involved in *recA*-mediated HR events (such as *ruvC*) and DNA repair by the error-prone TLS pathway [87].

In summary, DNA repair pathways appear to operate in a similar way in PCC9511 than in well studied, model organisms such as *E. coli *or *Bacillus subtilis*. The signal, if any, that activates the DNA repair pathways in this organism is still unclear, however. If it operates through a photoreceptor, we predict that it involves a visible light sensor rather than a UV sensor. Indeed, there is some evidence for the presence of a blue light photoreceptor in *P. marinus *MED4 [88]. It must be noted that in the field, UV irradiation is always accompanied by high photon fluxes of visible light, so given its minimalist regulation system, it is quite possible that *Prochlorococcus *has only one light signalling pathway for both stresses. Alternatively, DNA repair mechanisms could be activated by reactive oxygen species that are produced in response to both stresses [89]. Further biochemical studies are needed to check which of our different hypotheses for the observed delay in S phase is the most likely.

## Methods

### Strain and culture conditions

The axenic *Prochlorococcus marinus *strain PCC9511 used in this study has a morphology, pigment content and 16S rRNA sequence identical to the fully sequenced strain MED4, a.k.a. CCMP1378 or CCMP1986 [90] and these strains are genetically extremely similar, if not identical. Cultures of PCC9511 were grown at 22 ± 0.5°C in 0.2 μm filtered PCR-S11 medium [90]. For all experiments using a modulated 12 h/12 h L/D cycle, we used a custom-designed, computer-controlled illumination system (hereafter called 'cyclostat'), a modification of a previously described system [91] with an UV module added. PAR was provided by two symmetrical banks of 8 dimmable, U-shaped Philips PL-L 90 daylight fluorescence tubes (Philips Lightning, Eindhoven, NL) located on each side of the 50 L glass tank containing the culture flasks, whereas UV radiation was supplied by five pairs of UVA-340 fluorescent tubes (Q-Panel Lab products, Westlake, OH, USA) located above the cultures. PAR level was adjusted to reach a midday maximum of 100 μmol photons m^-2 ^s^-1 ^for LL conditions and 900 μmol photons m^-2 ^s^-1 ^for HL conditions. For long or short term UV experiments, HL conditions were supplemented by a 12 h/12 h L/D cycle of UV radiation reaching 7.59 W m^-2 ^UVA (320-400 nm) and 0.57 W m^-2 ^UVB (280-320 nm) at virtual noon (see additional file 1: Fig. S1).

For preliminary growth experiments, replicate 600 mL batch cultures were maintained in 1L Erlenmeyer glass flasks (Schott Duran, Mainz, Germany) for HL only experiments or 1 L Erlenmeyer quartz flasks (Atelier Jean Premont, Bordeaux, France) for HL+UV experiments. For transcriptomic analyses, two 7 L replicate cultures were kept in exponential growth phase at cell densities of around 10^8 ^cells mL^-1 ^by continuous dilution with fresh medium, at a rate adjusted to population growth (e.g., 4.83 L must be added per day to a 7 L culture growing at one division per day). For these large-scale experiments, we used custom-made, cylindrical 8 L quartz flasks (Ellipse, La Chapelle-la-Reine, France). All cultures were acclimated to experimental light conditions at least two weeks before the start of sampling. For long-term HL+UV conditions, cultures were slowly acclimated by incrementally increasing the UV dose by ca. 2 W m^-2 ^steps with at least 2-3 days of acclimation at each step. To further reduce UV stress, the pre-cultures were diluted daily at dawn and maintained at a cell density higher than 5×10^5 ^cells ml^-1^. To check for the eventual occurrence of self shading, we analyzed the timing of the S phase peak and the percentage of cells in S in the peak in samples collected at different depths of the quarz flask (i.e. different distances from UV lamps) and observed that there were no significant differences (data not shown).

### Growth and cell cycle analyses by flow cytometry

Culture samples for cell density measurements and cell cycle analyses were taken automatically at 1 h intervals using an electronic peristaltic pump (Masterflex Cartridge Pump 8; Fisher Bioblock Scientific, Illkirch, France) fitted to a custom-designed fraction collector. Aliquots were kept at 4°C in the dark and fixation of cells was done within a maximum timeframe of 9 h after sampling, a delay shown to cause only negligible changes on the DNA content in *Prochlorococcus *cells [92]. 400 microliter aliquots were fixed in glutaraldehyde (0.25% final concentration; Sigma Aldrich, Saint-Louis, MO, USA), incubated for 10 min at 4°C in the dark, frozen in liquid nitrogen and stored at -80°C. Frozen samples were thawed at room temperature (RT), then diluted in TE buffer (pH 9) (Tris HCl 10 mM, EDTA 1 mM) and cell concentrations were analyzed in the presence of 0.95 μm fluorescent microspheres (Polysciences, Warrington, PA, USA) which were used as internal references as previously described [93]. For cell cycle analyses, diluted samples were first stained with SYBR Green I (Invitrogen Molecular Probes, Carlsbad, CA, USA), used at a final concentration of 10^-4 ^of the commercial stock solution, as described [94]. Samples were analyzed either on a BD FACS Aria or a BD FACS Canto flow cytometer (Becton Dickinson Biosciences, San Jose, CA, USA), both equipped with a blue (488 nm) excitation laser. Cell count data files were analysed using the CytoWin 4.31 software [95] (available at http://www.sb-roscoff.fr/Phyto/) and cell cycle data files using the MultiCycle 4.0 software suite (Phoenix Flow Systems, San Diego, CA, USA). The duration of particular cell cycle phases was estimated based on the equations of Carpenter and Chang [30]. For batch cultures, division rates per day were computed from cell number variations using: ; where *μ*_nb _ is the estimated growth rate (d^-1^) and *N(t) *is the average cell concentration of two duplicate cultures at time points *t_2 _*and *t_1 _*taken at a 24 h interval, in a period when no division occurred, e.g. early morning when most cells were in G_1 _phase. For continuous cultures, division rates were estimated from cell cycle data using the formula of Carpenter and Chang [30]: ; where *μ*_cc _is the estimated growth rate (d^-1^), *n *is the number of samples collected at fixed intervals during one diurnal cycle, *f_S _*(*t*_i_) and *f_G2 _*(*t*_i_) are the fractions of cells in S and G_2 _phases at time *t*_i_, *T*_S_+*T*_G2 _(h) is the sum of S and G_2 _phases durations, computed as twice the delay (Δ*t*) between the peaks of cells in these phases [2 × (*t*_G2max _- *t*_Smax_)].

### RNA sampling and extraction

For transcriptomic analyses, cultures were sampled by pumping 400 mL aliquots into 1 L glass Erlenmeyer flasks eight times per L/D cycle during three consecutive days, with a shortened sampling interval around the expected S phase period, i.e. at 06:00, 09:00, 12:00, 15:00, 18:00, 20:00, 22:00 and 02:00. Immediately after harvesting, samples were chilled by swirling into liquid nitrogen for about 30 s (so that their temperature rapidly dropped down to ca. 4°C) and transferred into pre-chilled 450 mL polycarbonate centrifuge buckets (Beckman Coulter, Fullerton, CA, USA) containing a Pluronic F68 solution (0.005% final concentration; Sigma Aldrich). Samples were then harvested by centrifugation at 17,700 × g for 7 min at 4°C followed by a second centrifugation in microtubes (1.5 min at RT and 16,100 × g). Cell pellets were finally re-suspended in 500 μl Trizol (Invitrogen, Carlsbad, CA, USA), frozen in liquid nitrogen and kept at -80°C. During all transfer steps, samples were kept on ice in the dark. The total workflow from sampling to freezing the samples took no longer than 18 min.

RNA extractions were performed mainly as described in [45] except that the miRNeasy kit was used (as recommended by the manufacturers; Qiagen, Valencia, CA, USA) instead of the RNeasy protocol (after Trizol extraction), in order to recover both large and small RNAs. Two successive DNase treatments were performed on the columns using the Qiagen RNase-free DNase Set (Qiagen), followed by elution from the column in 30 ml DEPC-treated water. RNA samples were precisely quantified using a NanoDrop 1000 spectrophotometer (Thermo Scientific, Wilmington, DE, USA), quality-controlled with a BioAnalyzer 2100 using the RNA 6000 Nano Kit (Agilent, Santa Clara, CA, USA) and the absence of significant DNA contamination was confirmed by qPCR. All RNA samples were frozen in liquid nitrogen and stored at -80°C.

### Quantitative PCR analyses

Real time qPCR analyses were carried out on a DNA Engine/Chromo4 Real Time PCR-Detector (BioRad, Hercules, CA, USA) and using absolute SYBR Green ROX Mix (Abgene, Epsom, UK), as previously described [47] but starting from 50 ng of each RNA sample and using 250 nM of each primer. Primers used in this study, which were designed using Primer-Express software (v2.0; Applied Biosystems, Foster City, CA, USA), are listed in Table 4. All reactions were performed in duplicate. Resulting data were analysed using the comparative CT (ΔΔCT) method as described in the Applied Biosystems user bulletin #2 [96] using the *rnpB *gene as an internal standard [27] to normalize the transcript levels from all time points and light conditions to the HL 06:00 time point, which was used as a reference.

**Table 4:** Primer sets used for qPCR analyses of the diel cycle of expression of selected genes from *Prochlorococcus marinus *PCC9511.

Locus tag	Gene	Forward Primer	Reverse Primer
RNA_42	*rnpB*	64-AAGTCCGGGCTCCCATATG	135-TGTGGCACTATCCTCACGGTTA
PMM0285	*phrA*	1000-GGAGAGACCGGAGTACCTATAGTTGA	1088-GCGACTATCATCCTACATCTATTATGCA
PMM0395	*sepF*	397-GAAAGAGTCGGTGAAAGCATTTTT	470-GCCTCCTCTGGGAAAGAACTAGT
PMM0565	*dnaA*	492-TGGTGTCGGTCTTGGAAAGAC	568-CTTTCGCATCTGGATCAATTTCT
PMM0937	*umuC*	762-TCAAGTAAGTAGAAGCTTTGGAAAACC	889-TAGTAATGGCAGATGATTTTAAGCTTTG
PMM1054	*ruvC*	331-GCAGGCTCTGGCAAAGCA	407-TTTGGTGCACGGGTTAAATTT
PMM1077	*sasA*	430-CTTTTAAGAATGGTTGCACATGAATT	511-TTTGTCCTAGTTTTTGACTTTGAATAGC
PMM1262	*lexA*	227-AGATCTTTGAGGGAGTCCCAATT	299-TGGAGGTCGGAAAATGTTTCA
PMM1309	*ftsZ*	415-GGGATAGTAACCAAGCCATTTTCA	494-TCTGCTAATCTTGCAATCCCTTCT
PMM1342	*kaiC*	666-AGGAACCGTACATATGAAAGGAGAA	746-CTCATCGCTCCTAAGGCAAAA
PMM1343	*kaiB*	142-AAACAACCTCAACTTGCTGAAGAA	193-TAACAGGAGGAGGTAAAATCTTTGC
PMM1452	*atpD*	435-AATCGACCCATCCCTCATTG	512-ATTTGAGAGGCAAGACTAGCATCA
PMM1455	*atpH*	68-GCCCGGGCCTTGGA	129-AGGTTGGCGGGCAATACC
PMM1562	*recA*	319-GCTGAACATGCTTTAGATCCAGTCT	398-GTATCTGGCTGCGAAACTAGTAAATTT
PMM1629	*rpoD8*	726-CAAAGCTGGGCAGCCAGTA	820-CAGTCCATTTCGATTTGCTCATC
PMM1645	*mutS*	2281-ACAAGAATTGGAGCCGTAGATGA	2366-TGATTTAGTATTGATGCTGTTTCTGACA
PMM1697	*rpoD4*	746-CTCAAAGTGCTCCATGCGC	848-GATTGTTCTATCCATCCCTTCCA
PMM1712	*uvrA*	2775-GATAATTGATCTTGGACCTGATGGA	2865-ACTTATTGGATGCTTCGCAACA

The locus tag of the corresponding genes in *P. marinus *MED4 are indicated

### DNA microarray analyses

Microarray analyses were performed for time points 15:00, 18:00, 20:00 and 22:00 in HL and HL+UV conditions for two L/D cycles and two culture replicates, resulting in a total of 4 biological replicates per time point and light condition. All microarray expression analyses described in this study were performed using a *P. marinus *MED4 whole genome 4-Plex tiling microarray (Roche NimbleGen, Madison, WI, USA) carrying 4 × 60,053 probes with average size of 50 nucleotides (assuming that the genome of *P. marinus *PCC9511 is identical to that of MED4). cDNA labeling and hybridization steps were performed as recommended by the manufacturer [97]. Briefly, cDNA was synthesized from 10 μg of total RNA using the SuperScript™ Double-Stranded cDNA Synthesis kit (Invitrogen, Carlsbad, CA, USA) followed by cDNA labeling of 1 μg of double stranded cDNA using 5'-Cy3- or 5'-Cy5-labeled random primers (TriLink Technologies, San Diego, CA, USA). cDNA amplification and labeling efficiency was checked using the NanoDrop ND-1000 spectrophotometer, a minimum of a 10-fold cDNA increase being considered necessary for further use of the sample. Subsequent hybridization of labeled cDNA (2 μg of each labeled cDNA diluted in Nimblegen hybridization solution) to the NimbleGen array was performed overnight (16 h at 42°C in the dark) using the NimbleGen Hybridization System. Array slides were washed and dried using NimbleGen Wash Buffer kit, followed by scanning using the GenePix Personal 4100A scanner (Molecular Devices, Sunnyvale, CA, USA) at 5 μm resolution. The NimbleScan v2.6 software suite [98] was then used to extract the raw probe signal intensities for both Cy3 and Cy5 channels from the array TIFF images. In order to maximize the number of spots with a significant signal to background ratio, the reference sample hybridized on all arrays corresponded to a RNA pool of all samples of one complete day harvested in both light conditions and at all stages under investigation (all time points, cultures A and B, HL and UV conditions). Furthermore, replicate samples from the two examined L/D cycles (the same time point and light condition) were systematically hybridized in dye switch experiments in order to minimize bias due to differential dye bleaching or unequal incorporation of the Cy3 and Cy5 dyes during cDNA labeling reactions.

All microarray experiments were MIAME compliant and raw data were deposited under experiment name PCC9511-15-18-20-22 and accession number E-TABM-1028 at the ArrayExpress database of the EMBL-EBI (http://www.ebi.ac.uk/microarray-as/ae/).

### Statistical Analyses of microarrays

Statistical analyses were done using custom-designed scripts written under the R environment [99]. The probe dataset used in this study covered 1,968 probes out of the 2,014 genes identified so far in the MED4 genome [23,28]. The missing genes (see additional file 6: Table T2) corresponded to two probe categories that were systematically removed from the analysis. These probes were either to highly conserved multiple copy genes for which it was not possible to design specific probes (e.g. for some *hli *genes) or to very short ORFs for which the only designed probes were overlapping another gene or intergenic areas. The functional category of each gene was assigned using the Cyanobase database [100].

Microarray background bias was removed using the robust multi-chip average (RMA) background subtraction algorithm [101] from the preprocess Core R package implemented Bioconductor, an open source and open development software project [102]. This step was followed by normalization of the Cy3 and Cy5 signal intensities within arrays by loess normalization as well as between arrays by applying a quantile normalization, implemented in the R package LIMMA [103]. Data summarization of preprocessed probe sets covering individual genes was done by using the median polishing algorithm from the stats R package [99]. Student's t-test and the linear modeling features and empirical Bayes test statistics of the LIMMA package [104] were used to perform pairwise comparison of the different light conditions at the same time point (i.e. UV15 *vs*. HL15, UV18 *vs*. HL18, UV20 *vs*. HL20, UV22 *vs*. HL22) as well as comparing the S phase maximum under HL and UV (i.e. UV20 *vs*. HL18). Variance between all data points was also analyzed using one way ANOVA analysis and two way ANOVA analysis (TFA) where "light" and "time" were chosen to create suitable groups [105,106]. Since multiple tests were performed, statistical significance was adjusted based on the Benjamini and Hochberg algorithm [107] to control the FDR at 1%. Finally, to investigate the technical and biological reproducibility of our results, hierarchical clustering analyses [108] was performed with the hclust function from the stats R package [99] using the clustering method "average" and a Pearson correlation on a subset of differentially expressed genes selected based on the statistical significance of their differential expression as determined by one way ANOVA (FDR ≤ 0.1).

## Authors' contributions

LG, FP, DK and CK conceived the experiments. CK, FP, DMF, CB, NB, XL, PG and LG participated in sampling. CK did the flow cytometry measurements and cell cycle analyses. CK and MR extracted RNA samples and performed the microarrays and qPCR analyses. LG, GLC and MF wrote scripts in R to analyze microarrays and CK and MR participated in these analyses. JFL, LG and FP conceived and/or built the UV-visible cyclostat. CK, FP, DK and LG wrote the paper. All authors read and approved the final manuscript.

## Supplementary Material

Additional file 1 **Figure S1. Diel cycle of visible and UV radiations, as measured in the cyclostat growth chamber**. The different plots correspond to the photosynthetically active radiation [PAR; E_max_(400-700 nm) = 875 μmol photons m^-2 ^s^-1^; red line], the total UV radiation [E_max_(280-400 nm) = 8.22 W m^-2^; green line], the UV-A radiation [E_max_(320-400 nm) = 7.59 W m^-2^; yellow line] and the UV-B radiation [E_max_(280-320 nm) = 0.57 W m^-2^; violet line] components. When only visible light neon tubes were switched on, UV radiation levels were near detection limits [E_max_(280-400 nm) = 0.04 W m^-2^; data not shown]. Click here for file

Additional file 2 **Figure S2. Examples of flow cytograms and cell cycle analyses of *Prochlorococcus marinus *PCC9511 cells grown under HL and sampled at different times of the L/D cycle. A**, dot plot of green fluorescence from DNA *vs*. side scatter, for a culture sample taken during the G_1 _phase, stained with the DNA dye SYBR Green I, then analyzed by flow cytometry. **B**, FL1 histogram of the same sample as in Fig. A, showing the DNA frequency distribution of *Prochlorococcus *cells, from which the proportions of cells in G_1_, S and G_2 _phases were calculated using the *MultiCycle AV™ *software. **C**, same as graph A, but for a culture sample taken during the S phase. **D**, same as graph B for the sample used to draw graph C. **E**, same as graph A, but for a culture sample taken during the G_2 _phase. **F**, same as graph B for the sample used to draw graph E. Click here for file

Additional file 3 **Table T1. Complete set of gene expression data as measured by microarray analyses**. This table includes locus tags, gene names, product description as well as cyanobase functional categories and sub-categories for all 1,963 genes present on the PCC9511 array. Expression data are shown as log_2_(FC) calculated for each experimental sample (blue background) as well as for the 5 pairwise comparisons performed in this study (UV15 *vs*. HL15, UV18 *vs*. HL18, UV20 *vs*. HL18, UV20 *vs*. HL20 and UV22 *vs*. HL22; green background). For the latter, p-values and adjusted p-values were calculated using LIMMA and t-test (beige background). Values highlighted in red correspond to genes and pairwise comparisons for which adjusted p-values (FDR) was ≤ 0.1 and log_2_(FC) > 1. This subset of genes corresponds to the one used to build Fig. 4. The last columns show p-values and adjusted p-values calculated with one-way and two-way ANOVA where group 1 corresponds to light treatment and group 2 to "sampling time" (purple background). Click here for file

Additional file 4 **Figure S3. Patterns of *atpD *and *atpH *gene expression of L/D-synchronized *Prochlorococcus marinus *PCC9511 cultures under HL and UV growth conditions, as measured by qPCR**. The percentage of cells in the S phase of the cell cycle under HL (solid line) and HL+UV (dashed line) are also shown for comparison. Error bars indicate mean deviation for two biological replicates. Grey and black bars indicate light and dark periods. Click here for file

Additional file 5 **Figure S4. Sequence alignment of LexA homologs**. LexA protein sequences from *Prochlorococcus marinus *MED4 (PMM1262), *Synechococcus *sp. WH7803 (SynWH7803_1680) and *Synechocystis *sp. PCC6803 (Sll1626) were aligned against the *Escherichia coli *K12 LexA sequence (B4043). The DNA binding domain, preventing expression of DNA repair proteins (blue frame) and the peptidase S24-like domain, catalyzing self-cleavage of LexA (green frame) are indicated as well as conserved bases involved in the LexA repressor cleavage reaction (A84-G85 cleavage bond, S119 nucleophile, basic K156; red frame; [80]. Sequence alignments were made with BioEdit using ClustalW. Click here for file

Additional file 6 **Table T2**. Subset of *P. marinus *PCC9511 genes not included in microarray analyses. Click here for file
