# Supplementary figures and images for: Ultraviolet stress delays chromosome replication in light/dark synchronized cells of the marine cyanobacterium Prochlorococcus marinus PCC9511
Source: BMC Microbiol. 2010 Jul 29;10:204. doi: 10.1186/1471-2180-10-204 (PMC2921402; doi:10.1186/1471-2180-10-204)

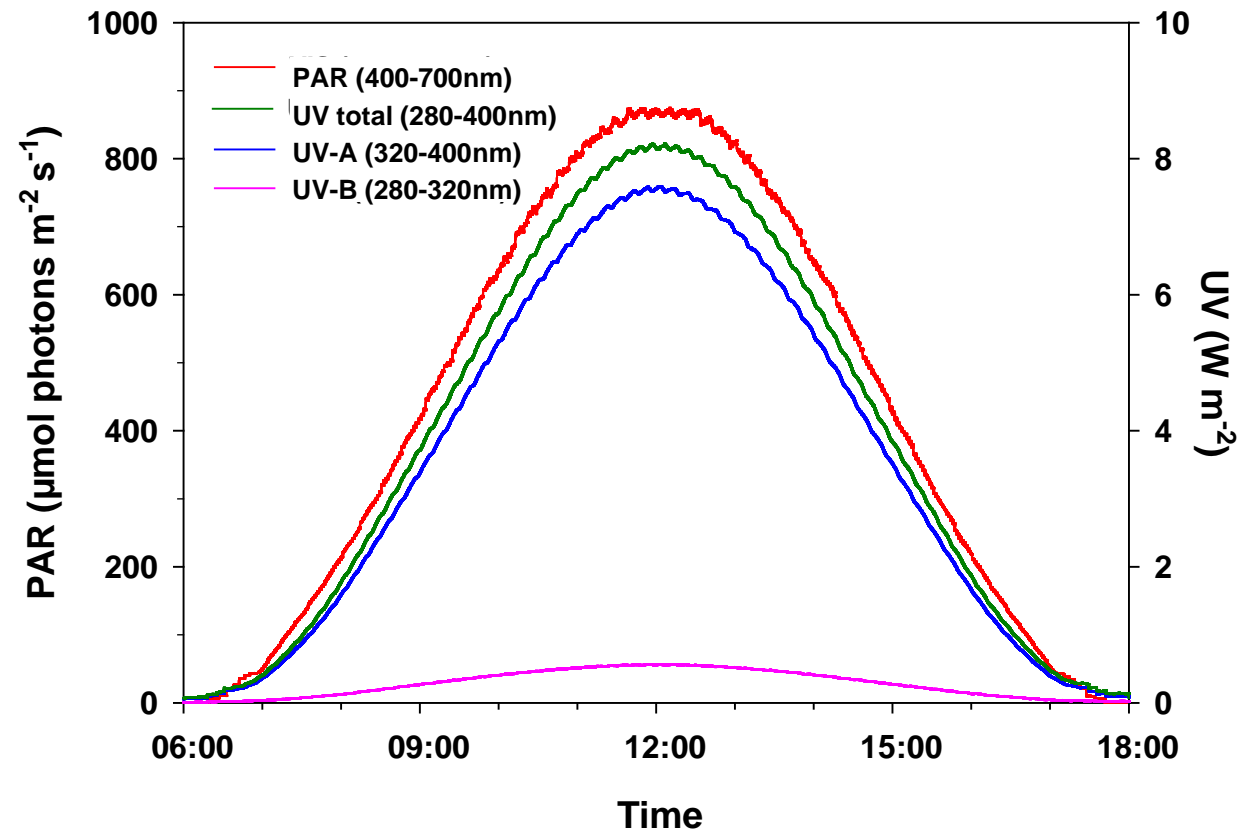

Supplement: Additional file 1 — Figure S1. Diel cycle of visible and UV radiations, as measured in the cyclostat growth chamber. The different plots correspond to the photosynthetically active radiation [PAR; Emax(400-700 nm) = 875 μmol photons m-2 s-1; red line], the total UV radiation [Emax(280-400 nm) = 8.22 W m-2; green line], the UV-A radiation [Emax(320-400 nm) = 7.59 W m-2; yellow line] and the UV-B radiation [Emax(280-320 nm) = 0.57 W m-2; violet line] components. When only visible light neon tubes were switched on, UV radiation levels were near detection limits [Emax(280-400 nm) = 0.04 W m-2; data not shown]. [file 1471-2180-10-204-S1.PDF]

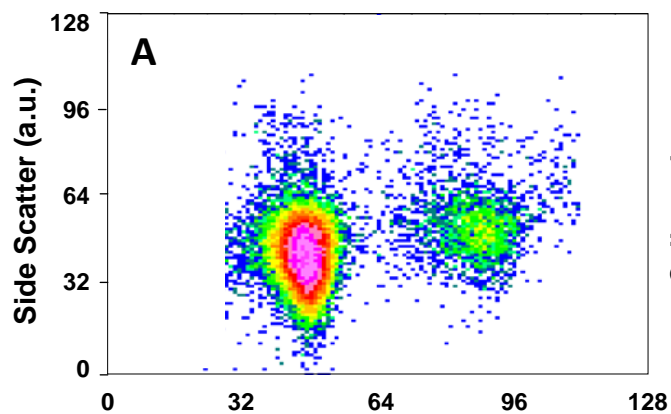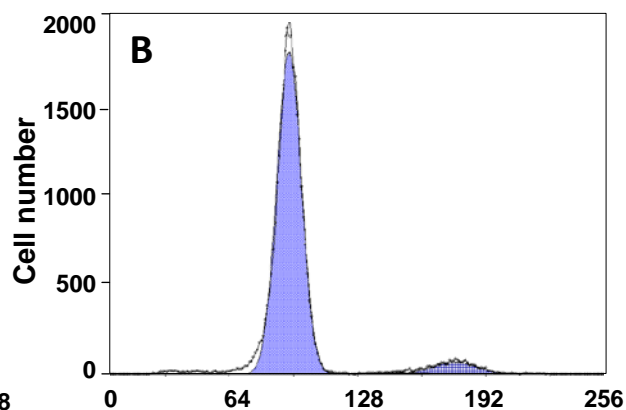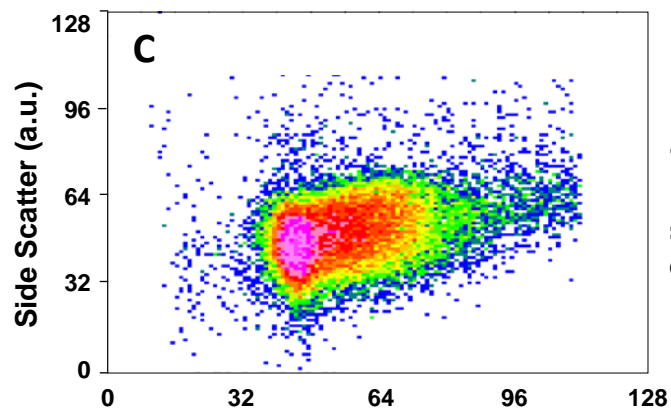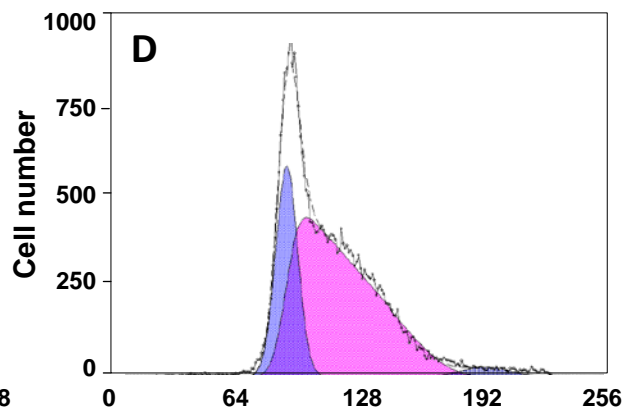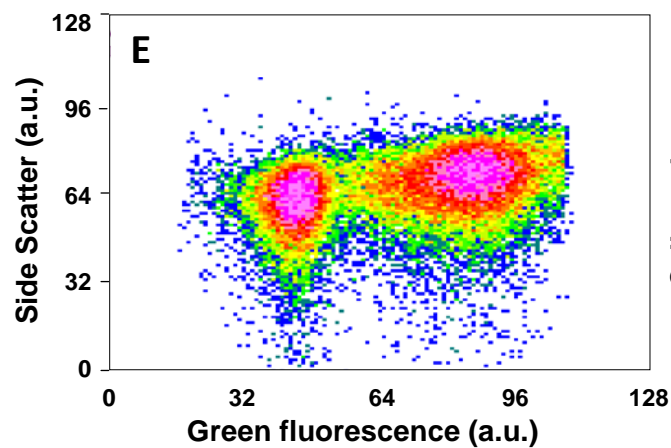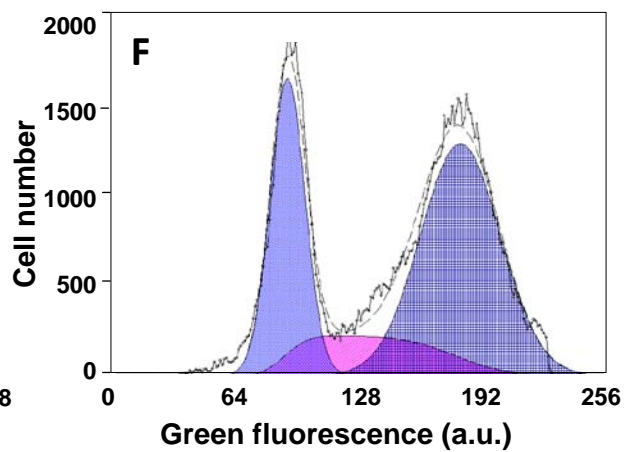

Supplement: Additional file 2 — Figure S2. Examples of flow cytograms and cell cycle analyses of Prochlorococcus marinus PCC9511 cells grown under HL and sampled at different times of the L/D cycle. A, dot plot of green fluorescence from DNA vs. side scatter, for a culture sample taken during the G1 phase, stained with the DNA dye SYBR Green I, then analyzed by flow cytometry. B, FL1 histogram of the same sample as in Fig. A, showing the DNA frequency distribution of Prochlorococcus cells, from which the proportions of cells in G1, S and G2 phases were calculated using the MultiCycle AV™ software. C, same as graph A, but for a culture sample taken during the S phase. D, same as graph B for the sample used to draw graph C. E, same as graph A, but for a culture sample taken during the G2 phase. F, same as graph B for the sample used to draw graph E. [file 1471-2180-10-204-S2.PDF]

Normalized expression (Fold change)

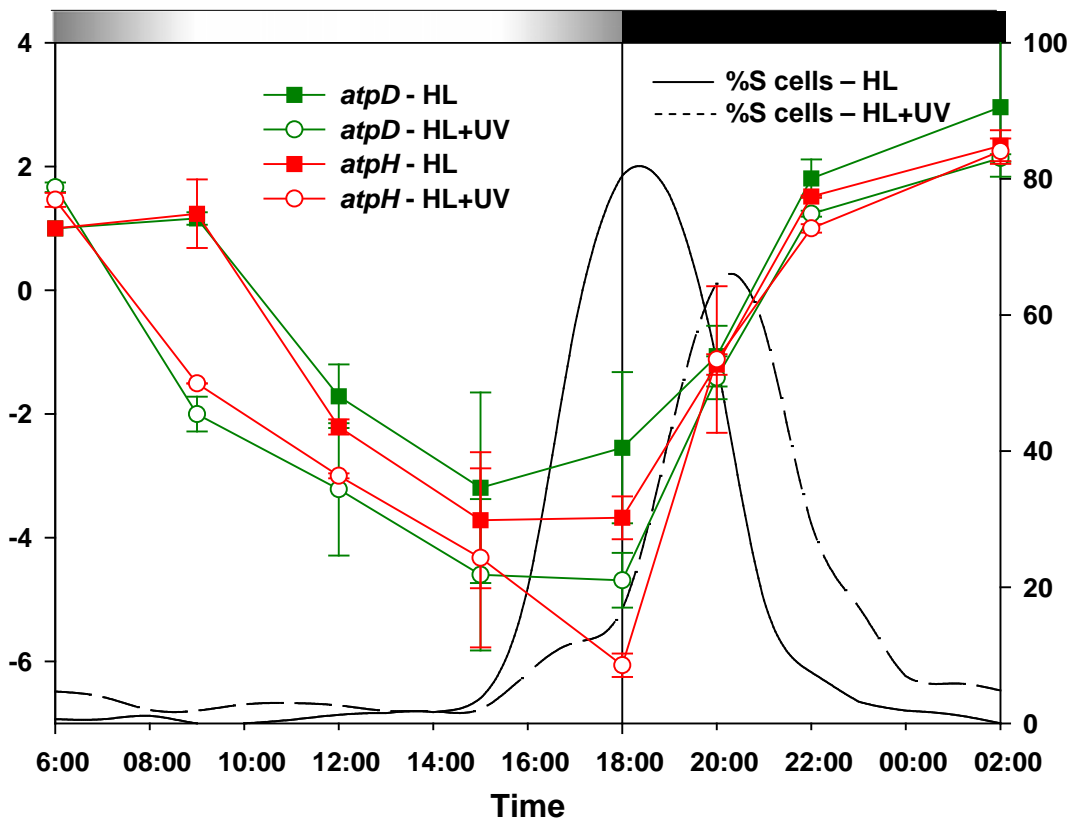

Cells in S phase (%)

Supplement: Additional file 4 — Figure S3. Patterns of atpD and atpH gene expression of L/D-synchronized Prochlorococcus marinus PCC9511 cultures under HL and UV growth conditions, as measured by qPCR. The percentage of cells in the S phase of the cell cycle under HL (solid line) and HL+UV (dashed line) are also shown for comparison. Error bars indicate mean deviation for two biological replicates. Grey and black bars indicate light and dark periods. [file 1471-2180-10-204-S4.PDF]
