# Supplementary material for: Ultraviolet stress delays chromosome replication in light/dark synchronized cells of the marine cyanobacterium Prochlorococcus marinus PCC9511
Source: BMC Microbiol. 2010 Jul 29;10:204. doi: 10.1186/1471-2180-10-204 (PMC2921402; doi:10.1186/1471-2180-10-204)
Supplement: Additional file 5 — Figure S4. Sequence alignment of LexA homologs. LexA protein sequences from Prochlorococcus marinus MED4 (PMM1262), Synechococcus sp. WH7803 (SynWH7803_1680) and Synechocystis sp. PCC6803 (Sll1626) were aligned against the Escherichia coli K12 LexA sequence (B4043). The DNA binding domain, preventing expression of DNA repair proteins (blue frame) and the peptidase S24-like domain, catalyzing self-cleavage of LexA (green frame) are indicated as well as conserved bases involved in the LexA repressor cleavage reaction (A84-G85 cleavage bond, S119 nucleophile, basic K156; red frame; [80]. Sequence alignments were made with BioEdit using ClustalW. [file 1471-2180-10-204-S5.PDF]

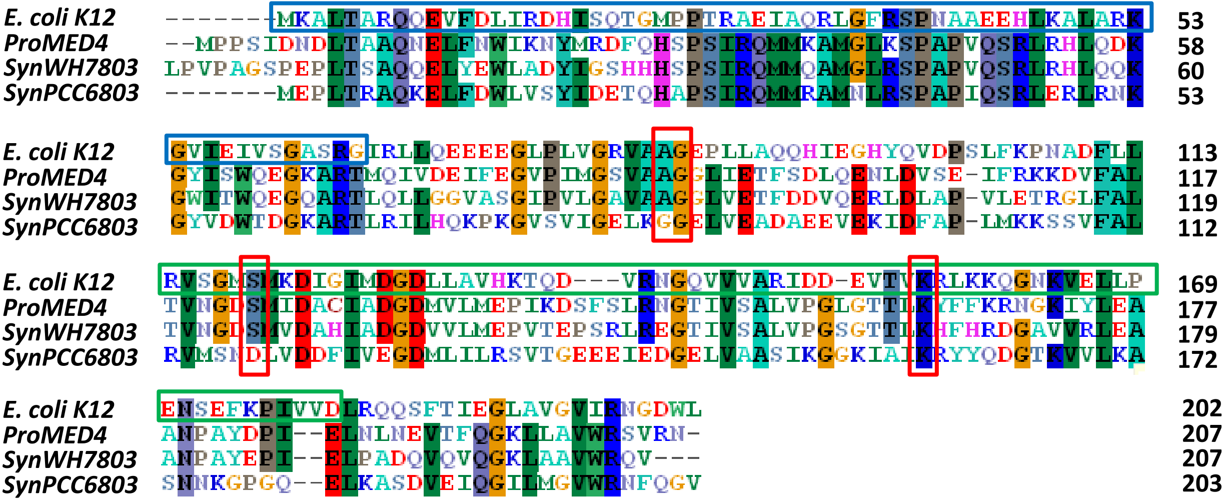
E. coli K12 -------MKALTARQQEVFDLIRDHISQTGMPPTRAEIAQRLGFRSPNAAEEHLKALARK 53
ProMED4 --MPPSIDNDLTAAQNELFNWIKNYMRDFQHSPSIRQMMKAMGLKSPAPVQSRLRHLQDK 58
SynWH7803 LPVPAGSPEPLTSAQQELYEWLADYIGSHHHSPSIRQMMQAMGLRSPAPVQSRLRHLQQK 60
SynPCC6803 -------MEPLTRAQKELFDWLVSYIDETQHAPSIRQMMRAMNLRSPAPIQSRLERLRNK 53
E. coli K12 GVIEIVSGASRGIRLLQEEEEGLPLVGRVAAGEPLLAQQHIEGHYQVDPSLFKPNADFLL 113
ProMED4 GYISWQEGKARTMQIVDEIFEGVPIMGSVAAGGLIETFSDLQENLDVSE-IFRKKDVFAL 117
SynWH7803 GWITWQEGQARTLQLLGGVASGIPVLGAVAAGGLVETFDDVQERLDLAP-VLETRGLFAL 119
SynPCC6803 GYVDWTDGKARTLRILHQKPKGVSVIGELKGGELVEADAEEVEKIDFAP-LMKKSSVFAL 112
E. coli K12 RVSGMSMKDIGIMDGDLLAVHKTQD---VRNGQVVVARIDD-EVTVKRLKKQGNKVELLP 169
ProMED4 TVNGDSMIDACIADGDMVLMEPIKDSFSLRNGTIVSALVPGLGTTLKYFFKRNGKIYLEA 177
SynWH7803 TVNGDSMVDAHIADGDVVLMEPVTEPSRLREGTIVSALVPGSGTTLKHFHRDGAVVRLEA 179
SynPCC6803 RVMSNDLVDDFIVEGDMLILRSVTGEEEIEDGELVAASIKGGKIAIKRYYQDGTKVVLKA 172
E. coli K12 ENSEFKPIVVDLRQQSFTIEGLAVGVIRNGDWL 202
ProMED4 ANPAYDPI--ELNLNEVTFQGKLLAVWRSVRN- 207
SynWH7803 ANPAYEPI--ELPADQVQVQGKLAAVWRQV--- 207
SynPCC6803 SNNKGPGQ--ELKASDVEIQGILMGVWRNFQGV 203
